# Role of the DEAD-box RNA helicase DDX5 (p68) in cancer DNA repair, immune suppression, cancer metabolic control, virus infection promotion, and human microbiome (microbiota) negative influence

**DOI:** 10.1186/s13046-023-02787-x

**Published:** 2023-08-19

**Authors:** Fengzhi Li, Xiang Ling, Sayan Chakraborty, Christos Fountzilas, Jianmin Wang, Anmbreen Jamroze, Xiaozhuo Liu, Pawel Kalinski, Dean G. Tang

**Affiliations:** 1 Department of Pharmacology & Therapeutics, Roswell Park Comprehensive Cancer Center, Elm and Carlton Streets, Buffalo, NY 14263 USA; 2 Program of Developmental Therapeutics, Roswell Park Comprehensive Cancer Center, Buffalo, NY 14263 USA; 3 Canget BioTekpharma LLC, Buffalo, NY 14203 USA; 4 Department of Medicine, Roswell Park Comprehensive Cancer Center, Buffalo, NY 14263 USA; 5 Department of Bioinformatics & Biostatistics, Roswell Park Comprehensive Cancer Center, Buffalo, NY 14263 USA; 6 Department of Immunology, Roswell Park Comprehensive Cancer Center, Buffalo, NY 14263 USA; 7 Program of Tumor Immunology & Immunotherapy, Roswell Park Comprehensive Cancer Center, Buffalo, NY 14263 USA

**Keywords:** DDX5, p68, DNA repair, Immune suppression, Virus infection promotion, Cancer metabolic control, Microbiota negative influence, Oncotarget, Cancer therapeutics, DDX5 degrader FL118

## Abstract

There is increasing evidence indicating the significant role of DDX5 (also called p68), acting as a master regulator and a potential biomarker and target, in tumorigenesis, proliferation, metastasis and treatment resistance for cancer therapy. However, DDX5 has also been reported to act as an oncosuppressor. These seemingly contradictory observations can be reconciled by DDX5’s role in DNA repair. This is because cancer cell apoptosis and malignant transformation can represent the two possible outcomes of a single process regulated by DDX5, reflecting different intensity of DNA damage. Thus, targeting DDX5 could potentially shift cancer cells from a growth-arrested state (necessary for DNA repair) to apoptosis and cell killing. In addition to the increasingly recognized role of DDX5 in global genome stability surveillance and DNA damage repair, DDX5 has been implicated in multiple oncogenic signaling pathways. DDX5 appears to utilize distinct signaling cascades via interactions with unique proteins in different types of tissues/cells to elicit opposing roles (e.g., smooth muscle cells versus cancer cells). Such unique features make DDX5 an intriguing therapeutic target for the treatment of human cancers, with limited low toxicity to normal tissues. In this review, we discuss the multifaceted functions of DDX5 in DNA repair in cancer, immune suppression, oncogenic metabolic rewiring, virus infection promotion, and negative impact on the human microbiome (microbiota). We also provide new data showing that FL118, a molecular glue DDX5 degrader, selectively works against current treatment-resistant prostate cancer organoids/cells. Altogether, current studies demonstrate that DDX5 may represent a unique oncotarget for effectively conquering cancer with minimal toxicity to normal tissues.

## Introduction

Over the last 3 decades, the DEAD-box RNA helicase (RH) family proteins have been isolated from a wide range of organisms from viruses to E. coli to humans [1]. Their mechanisms of action, functional diversity, regulation and potential targets for disease treatment have gradually emerged over time [2–13].

**Fig. 1:**
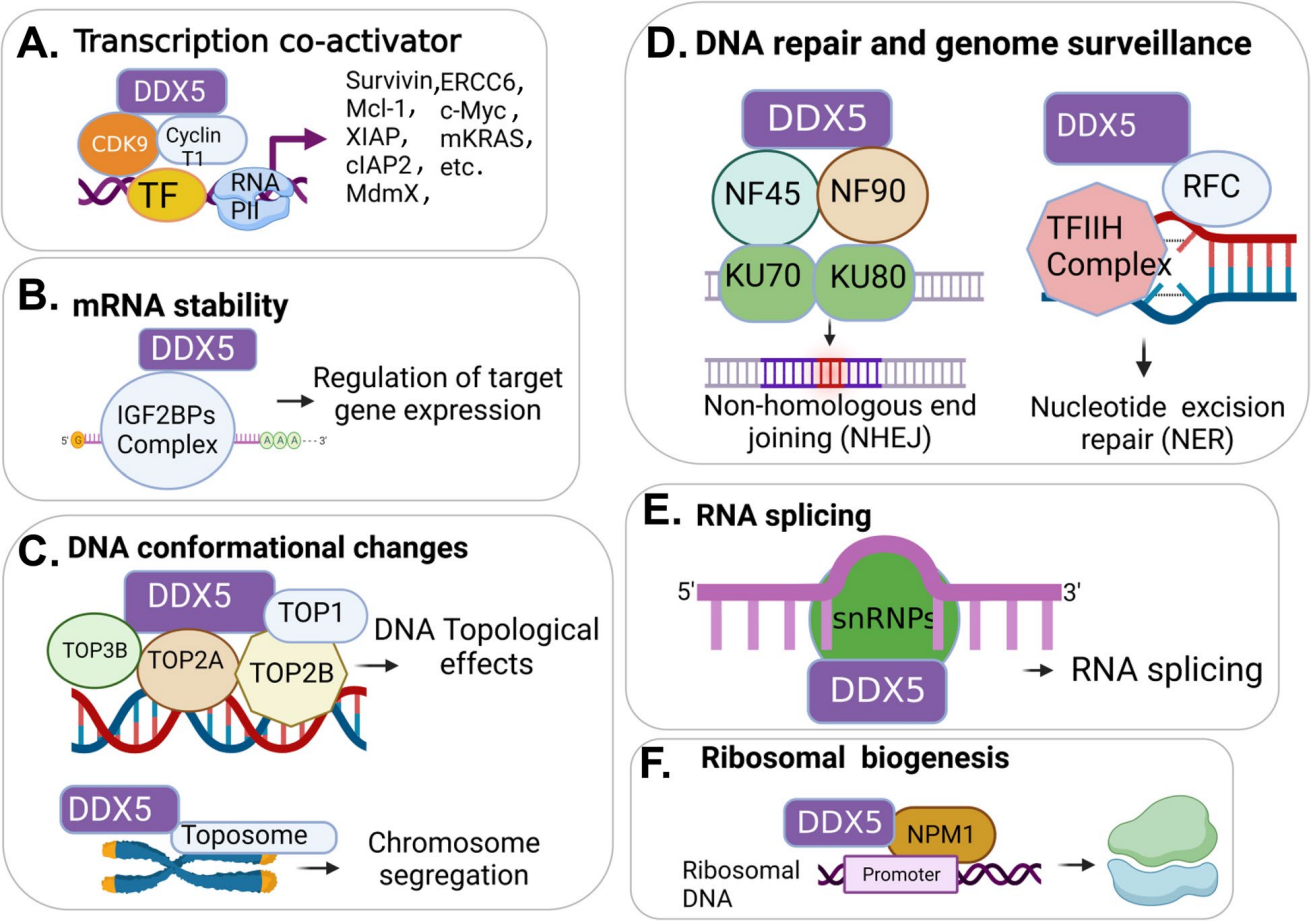
As a TF co-activator and an RNA helicase, DDX5 (p68) has diverse functions to act as an oncogenic master regulator: **A** DDX5-mediated transcriptional activation of many oncogenic genes by interacting with different TFs (e.g., c-Myc) together with CDK9/cyclin T1 complex, which bridges basic transcription machinery on the oncogene promoters; **B** DDX5 regulation of mRNA stability by interacting with RNA stability modulators (e.g., interacting with the IGF2BPs complex); **C** DDX5-regulated DNA conformational changes. This is achieved (i) through interaction with various topoisomerases (TOPs) to alter DNA topological structure, and (ii) through interaction with toposome (e.g., interacting with topoisomerase 2α-containing complex) for chromosome segregation; **D** DDX5 interaction with DNA repair regulators (Table 1) and involvement in DNA repair and genome surveillance. For example, DDX5 participates in NHEJ DNA repair by interacting with NF45/NF90 and Ku70/Ku80 complexes, and DDX5 participates in NER and facilitates DNA repair by interacting with replication factor C (RFC) proteins; **E** DDX5 regulation of the splicing of pre-RNA, microRNA (miRNA) and circular RNA (circRNA; e.g., Wang et al., Aging US 2023, 15:2525–40); and **F** DDX5 regulation of ribosome biogenesis through interacting with other regulators such as nucleophosmin 1 (NPM1) on the ribosomal DNA promoter. Note: The diverse functions of DDX5 presented herein were based on many literature reports but primarily based on the recent publication from Le et al., Mol Ther 2023 Feb 1; 31(2):471–486

While RHs such as eukaryotic translation initiation factor 4A1 (eIF4A1, also called DDX2A1), eIF4A2 (DDX2B), IF4AIII (DDX48), Ded1 (yeast ortholog of DDX3), and Dbp5 (yeast homolog of DDX19), have defined physiologic functions, the physiologic roles of most RHs are poorly understood [8, 12]. On the other hand, many RHs are known to be involved in human diseases especially in cancer [14, 15] and viral infections [16, 17]. In this review, we focus on DDX5, which is known to be a major member in the DEAD-box RH family, plays diverse roles in human cancer (Fig. 1), and potentially represents a prime target for cancer therapeutics [18–20]. Specifically, in this review we will discuss the diverse roles of DDX5 in DNA repair, immune suppression, cancer metabolic control, virus infection promotion, and negatively impacting microbiota, which in aggregate, support the notion that DDX5 is a master oncogenic regulator.

### DDX5 in genome stability surveillance and DNA repair

A general role of DEAD-box RHs in genome stability and DNA repair was recently reviewed [21]. Here, we focus on DDX5 and its role in genome stability surveillance and DNA repair (Fig. 1D) in more detail from a cancer therapeutic point of view and with an attempt to unify the seemingly contradictory observation of the oncogenic versus oncosuppressive functions of DDX5. We first elaborate on the role of DDX5 in genome stability and DNA repair and then review its role in R-loop resolution during DNA transcription and replication.

#### DDX5 in general DNA damage repair and cancer malignancy

DDX5 (p68) acts as a coactivator of the transcription factor (TF) p53 (Fig. 1A) to activate the p21^WAF1^gene and subsequent cell-cycle arrest in cancer cells [22]. During the post-transcriptional maturation of several growth-suppressive miRNAs induced by p53, p53 needs to directly interact with p68 (DDX5) in the Drosha miRNA maturation complex; and p68/DDX5 knockdown (KD) decreases p53-Drosha association and abolishes growth-suppressive miRNAs’ maturation in response to DNA damage [23]. Thus, in this context, p53 interaction with p68/DDX5 also favors cell-cycle arrest after DNA damage. However, while p68/DDX5 depletion inhibits p53 and RNA Pol II binding to the p21^WAF1^ promoter for its transcriptional activation [22] and blocks the maturation of growth-suppressive miRNA [23], DDX5 depletion does not interfere with p53 and RNA Pol II binding to the promoters of pro-apoptotic genes *Bax* or *PUMA* for their activation [24], indicating that DDX5 depletion induces apoptosis (due to damaged DNA unrepaired) instead of cell arrest for DNA repair. Studies using an inducible *DDX5* knockout (KO) mouse model further demonstrated that *DDX5* KO inhibits p21^WAF1^ and increased sensitivity to γ-irradiation leading to increased apoptosis in the bone marrow [24]. Intriguingly, in response to DNA damage induced by genotoxic stimuli, p53-mediated apoptosis induced by amphiregulin (AREG) required AREG interaction with DDX5 [25]. This may be an alternative approach to inhibit the DDX5 DNA repair function for AREG induction of apoptosis, because it was found that the DNA DSB-repairing protein Ku associated with DDX5 [26, 27] (Fig. 1D). Additionally, it has been reported that the lncRNA SLC26A4-AS1 suppresses DNA repair and thyroid cancer metastasis through its interaction with DDX5 and promotion of DDX5 degradation by the E3 ligase TRIM25 [28].

Rocchi’s group identified a panel of DNA repair-relevant proteins as DDX5-interacting partners in prostate cancer (PCa) cell lines (LNCaP, DU145, PC-3) but not in the normal prostatic cell line PNT1A (Table 1) [27]. This suggests that the interaction of DDX5 with various DNA repair proteins potentially plays a surveillant role in various types of DNA repair to maintain global genome stability and enhance therapy resistance, especially in androgen receptor-negative and castration-resistant PCa (CRPC) DU145 and PC-3 cells (Table 1) [27]. Furthermore, DDX5 KD decreased the DNA repair efficiency and sensitized DU145 cells towards irradiation or cisplatin treatment [27], indicating that DDX5 promotes resistance to treatment.

**Table 1:** DDX5 interacting proteins implicated in the DNA damage response identified in PCa cell lines versus normal prostatic cell line PNT1A^b^

**Proteins**	**PNT1A**	**LNCaP**	**DU-145**	**PC-3**	**DDR-related pathways**
XRCC6			x	x	NHEJ^a^
XRCC5			x	x	NHEJ
GTF2H4			x	x	NER
GTF2H1			x	x	NER
ERCC3			x	x	NER
GTF2H2			x	x	NER
GTF2H3			x	x	NER
RFC5		x	x	x	NER
UPF1			x	x	Unclear
PRPF19			x	x	NER, NHEJ
IGHMBP2			x	x	Unclear
MSH6		x	x		MR
TP53			x		BER, NER, DSBR, mitochondrial DNA repair
LIG3			x		BER, NER, alNHEJ, HR, mitochondrial DNA repair
RFC3			x		NER
ERCC2			x		NER
ERCC6				x	NER
RFC1				x	NER
TOP2A				x	DDR
RECQL4				x	HR
MRPS26				x	Mitochondrial DNA repair
MRPS35				x	Mitochondrial DNA repair
DTX3L				x	NHEJ

^a^*DDR* DNA damage response, *DSBR* double-strand break repair, *NHEJ* non-homologous end joining, *HR* homologous recombination, *MR* mismatch repair, *BER* base excision repair, *NER* nucleotide excision repair, *alNHEJ* alternative NHEJ

^b^Adapted from Le et al., Mol Ther. 2023 Feb 1; 31(2):471–486

The above studies highlight the key function of DDX5 as the regulator of the balance of p53-mediated cell growth arrest (for DNA repair) [22, 23] versus apoptosis [24–28]. Consistent with this conclusion, cells transfected with DDX5 siRNA were observed to have higher basal levels of apoptosis prior to the doxycycline-mediated induction of p53 expression [22]. Together, these observations suggest that targeting DDX5 could potentially shift cancer cells from a growth-arrested state (necessary for DNA repair) to apoptosis and cell killing.

#### Role of DDX5 in the R-loop resolution-involved DNA repair

DDX5 has emerged as a critical regulator in resolving DNA transcription-replication-coupled R-loop formation resolution to prevent cancer cell death as a result of DNA double-strand breaks (DSBs). Below is our updated review in this area.

**Fig. 2:**
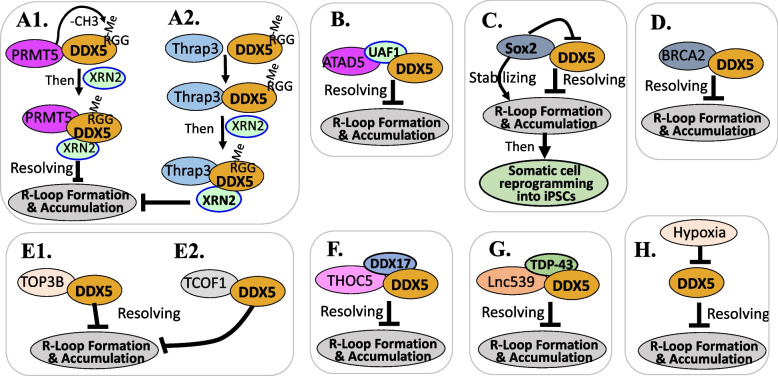
DDX5 plays a central role in resolving DNA replication / transcription-coupled DNA/RNA R-loop formation: **A1** PRMT5-mediated methylation of DDX5 on the arginine residue in RGG motif is required for DDX5 to recruit XRN2 to become a complex to resolve DNA/RNA R-loop formation. **A2** Thrap3 interaction with the RGG motif-methylated DDX5 to recruit XRN2 to resolve R-loop formation. **B** UAF1 is a mediator to form a ATAD5-UAF1-DDX5 complex to resolve R-loop formation. **C** Sox2 interacted with and inhibited DDX5 and thus stabilized R-loop formulation for somatic cell reprogramming into iPSCs. **D** BTAC2 interacts with and helps retain DDX5 to help DDX5 on DNA damage sites to resolve R-loop formulation. **E1** TOP3B interacts with DDX5 to resolve R-loop formation. **E2** TCOF1 interacts with DDX5 to resolve R-loop formation. **F** THOC5 recruits both DDX5 and DDX5 paralog DDX17 to resolve R-loop formation. **G** LncRNA Lnc530 recruits DDX5 and TDPJ-43 to prevent R-loop formation. **H** Hypoxia decreases DDX5 expression and thus, blocks DDX5 from accessing various forms of DNA

The aberrant transcription-associated R-loop formation causes catastrophic stresses during replication, resulting in genomic instability with DNA DSBs. Mersaoui et al. reported that DDX5 is a crucial player in the resolution of such R-loop formation in human osteosarcoma U2OS cells [29]. The study found that (i) arginine methyltransferase 5 (PRMT5) binds and methylates DDX5 at its RGG/RG motif, which is required for DDX5 interaction with 5'-3' exoribonuclease 2 (XRN2) and repression of cellular R-loops (Fig. 2A1); (ii) DDX5-deficient cells accumulate R-loops and cause spontaneous DNA DSBs and hypersensitivity to replication stress, and unrepaired DNA DSBs would induce apoptosis; (iii) DDX5 associates with XRN2 and resolves R-loops at transcriptional termination regions downstream of poly(A) sites, to facilitate RNA polymerase II (RNA Pol II) release and transcriptional termination [29].

The same group further defined a critical role for DDX5 in clearing R-loops at or near DSBs enabling proper DNA repair to avoid aberrations such as chromosomal deletions [30]. Specifically, the authors reported that (i) DDX5-deficient human osteosarcoma U2OS cells exhibited asymmetric end deletions on the side of the DSBs with significant overlap with a transcribed gene; (ii) DDX5 bound RNA transcripts near DSBs; (iii) DDX5 was excluded from DSBs in a transcription- and ATM activation-dependent manner; (iv) DDX5-deficient cells had increased R-loops near DSBs leading to delayed exonuclease 1 and replication protein A (RPA) recruitment to laser irradiation-induced DNA damage sites, resulting in homologous recombination repair defects [30]. These findings define a role of DDX5 in DNA repair by facilitating the clearance of RNA transcripts overlapping DSBs to ensure proper DNA repair.

Consistent with the finding that RGG motif-methylated DDX5 is required for its interaction with XRN2 [29], Kang et al. found that the thyroid hormone receptor-associated protein 3 (Thrap3) plays a causal role in promoting R-loop resolution via interaction with methylated DDX5 localized to R-loops in cancer cells [31]. These authors found that arginine methylation of DDX5 is required for its interaction with Thrap3, and the Thrap3-DDX5 axis induces the recruitment of XRN2 into the R-loops (Fig. 2A2) [31]. However, while these studies showed that shRNA silencing of Thrap3 increases R-loop accumulation and DNA damage, whether the silencing of DDX5 in this background would further increase R-loop accumulation and DNA damage is worthy of further exploration.

Kim et al. reported that multiple RHs (DDX1, DDX5, DDX21, DHX9) are involved in the proliferating cell nuclear antigen (PCAN) unloader ATAD5-mediated restriction of R-loop formation at the DNA replication fork [32]. However, studies from this report relevant to DDX5 [32] demonstrated that (i) DDX5 co-immunoprecipitated with ATAD5, UAF1, DHX9 and DDX21; (ii) the ubiquitin-specific protease 1 (USP1)-associated factor (UAF1) mediates interaction between ATAD5 and DDX5 (Fig. 2B); and (iii) consistent with UAF1 bridging ATAD5 and DDX5 interactions, simultaneous depletion of ATAD5 and DDX5 did not show synergistic or additive effects on R-loop increase, suggesting that ATAD5 and DDX5 regulate R-loop resolution in the same pathway [32]. These authors also found that DDX5’s DNA-RNA hybrid unwinding activity requires ATP hydrolysis as previously reported by Mersaoui et al. [29]. Together, these studies suggest that DDX5 plays a unique role in ATAD5-restricted R-loop formation.

Using mouse embryonic fibroblast (MEF) model, Li et al. [33] observed that (i) during somatic cell reprogramming to induced pluripotent stem cells (iPSCs), dynamic changes in R-loops were essential for reprogramming and occurred before gene expression changes; (ii) the stem cell TF Sox2 was the only factor in the Yamanaka cocktail (Oct4, Sox2, Klf4, c-Myc) that could overcome the inhibitory effects of RNaseH1 activity loss on reprogramming; (iii) DDX5 was a reprogramming barrier factor and Sox2 interacted with and inhibited DDX5 helicase activity-mediated R-loop–resolving capacity on R-loop sites and thus facilitated reprogramming, and the stabilization of R-loops by Sox2 is required for completing somatic cell reprogramming (Fig. 2C); (iv) a gradual increase in Sox2 rescued the reprogramming-inhibitory activities of DDX5 and DDX5 peaks were highly enriched at R-loops in iPSCs; and (v) Sox2 alone could not resolve either RNA/DNA hybrids or R-loops, but DDX5 alone exhibited a strong capacity to resolve these structures. Consistent with this observation, DDX5 inhibited R-loop levels of all pluripotent genes [33]. The authors concluded that (a) Sox2 plays an important role in reprogramming by ensuring the maintenance of R-loops and DDX5 acts as a barrier for reprogramming; (b) their findings support and reflect bivalent functions of DDX5 in regulating reprogramming, and (c) Sox2 itself does not resolve R-loops but prevents DDX5 from resolving R-loops [33]. The uniqueness of DDX5 appears not only in control of somatic cell reprogramming into iPSCs [33], but also in cancer cell model-based studies. Villarreal et al. [34] performed genome-wide R-loop mapping of DNA/RNA hybrid loci regulated by DDX5, XRN2, and PRMT5, and observed hundreds to thousands of R-loop gains and losses at transcribed loci in DDX5-, XRN2-, and PRMT5-deficient human osteosarcoma U2OS cells [34]. While DDX5, XRN2, and PRMT5 shared many R-loop gain loci at transcription termination sites, DDX5-depleted cells had unique R-loop gain peaks near the transcription start site that did not overlap with those in siXRN2 and siPRMT5-treated cells [34]. This suggests that DDX5 plays a unique role in transcription initiation that is independent of XRN2 and PRMT5.

Sessa et al. [35] report that (i) the tumor suppressor BRCA2 protein physically interacts with DDX5 in cancer cells (Fig. 2D); (ii) DDX5 depletion leads to a genome-wide accumulation of DNA-RNA hybrids particularly enriched at the DSB sites, which can be rescued by DDX5 overexpression in both DDX5-depleted cells and BRCA2-depleted cells [35]; (iii) BRCA2 helps retain DDX5 on DNA damage sites and stimulates R-loop-unwinding activity of DDX5; and (iv) DDX5-BRCA2 interaction favors DNA DSB repair by homologous recombination (HR) [35]. Thus, the study suggests that DDX5 appears to be a crucial master player in R-loop resolution through interactions with BRCA2 (Fig. 2D) [35].

Saha et al. [36] reported that (i) DNA topoisomerase 3β (TOP3B) is recruited to R-loop sites and plays a role in R-loop resolution; (ii) TOP3B interacts with DDX5, and this interaction does not appear to be mediated by DNA and to occur prior to R-loop induction by R-loop inducers (Fig. 2E1); (iii) either DDX5 depletion or TOP3B depletion would increase cellular R-loop levels but depletion of both DDX5 and TOP3B at the same time produced no further increase in R-loop levels [36], indicating their role in R-loop resolution to protect cancer cells from R-loop-induced damage through an epistatic manner (i.e., use the same pathway), similar to the case of simultaneous ATAD5 and DDX5 depletion [32]. Additionally, in gastric cancer, Nie et al. reported that (i) treacle ribosome biogenesis factor 1 (TCOF1) and DDX5 co-precipitate each other in a non-R-loop-mediated manner (Fig. 2E2); and (ii) depletion of TCOF1 and DDX5 at the same time does not further increase R-loops when compared to TCOF1 depletion alone, indicating their use of the same pathway [37]. However, whether TCOF1 stimulates the R-loop-unwinding activity of DDX5 requires further studies.

It is known that THO complex subunit 5 (THOC5, an mRNA export complex member) plays a key role in stem cell and cancer cell biology. Interestingly, a study from Polenkowski et al. reported that (i) THOC5 and THOC6 cooperate to remove harmful R-loops during transcription elongation by recruiting DDX5 and DDX5 paralog DDX17, but do not modulate the unwinding activity of DDX5/17 (Fig. 2F) [38]; (ii) cells with DDX5 and DDX17 depletion but not DDX50 and DXH15 depletion accumulate R-loops [38]; (iii) overexpression of DDX5 or DDX17 suppressed the R-loop accumulation in THOC5-depleted cells [38]; and (iv) THOC5-depleted cells showed a strong decrease of DDX5/17 recruited to the gene body but not gene promoters. Whereas, in contrast, R-loops significantly accumulated across the gene body of THOC5-dependent genes upon DDX5 or DDX17 depletion, suggesting that THOC5 is required for the recruitment of DDX5/17 during transcription elongation [38].

Gong et al. [39] observed that (i) a long noncoding RNA Lnc530 needs to recruit DDX5 and TDP-43 (TAR DNA-binding protein 43, a RNA/DNA-binding protein) to efficiently prevent R-loop formation in mouse embryonic stem cells (mESCs) (Fig. 2G); (ii) DDX5 KD has no effect on the expression of TDP-43 or Lnc530 but attenuates Lnc530-TDP-43 interaction, which can be restored by DDX5 re-expression in DDX5 KD cells. Similarly, TDP-43 KD attenuates Lnc530-DDX5 interaction. DDX5 can physically interact with TDP-43, and their interaction can be enhanced by R-loop induction, while DDX5-TDP-43 interaction significantly decreases in Lnc530 KD cells, indicating their interdependent interactions on R-loops; and (iii) DDX5 KD or TDP-43 KD in mESCs can induce both R-loop accumulation and higher levels of DNA DSBs, which can be alleviated by over-expression of RNase H1 or re-expression of respective protein, indicating that DDX5 and TDP-43 prevent R-loop accumulation and mediate the regulatory function of Lnc530 on R-loops in mESCs [39]. The ‘double insurance’ of such an efficient R-loop resolution strategy in normal ESCs may provide a great advantage to reduce potential toxicity in normal tissues and cells when DDX5 is used as a therapeutic target in cancer.

Additionally, Leszczynska et al. found that hypoxia decreases DDX5 expression and chromatin accessibility at gene promoters and impacts specific pathways [40]. This may include mRNA stability, DNA topological regulation, DNA repair, RNA splicing, and ribosome biogenesis (Fig. 1) as well as the R-loop interactome (Fig. 2H). Consistently, decreased DDX5 expression is associated with R-loops accumulation in cancer cells [40]. This finding suggests that DDX5 also plays a critical role in hypoxia response by increasing R-loop-associated DNA damage resulting from hypoxia-mediated inhibition of DDX5.

The literature discussions above [29–40] highlight the role of DDX5 as a center protein molecule and a partner for multiple other proteins to control R-loop formation/accumulation resolution and gene transcription, being involved in their individual signaling pathways in a very similar manner (Fig. 2A-H). Thus, targeting DDX5 instead of targeting its individual partners may be a particularly efficient way that is equivalent to target all partnering proteins in cancer therapy.

### DDX5’s DNA damage repair function in pancreatic and liver cancer

Given DDX5’s function in genome stability and DNA damage repair reviewed above, we will further expound on the DNA damage repair functions in two cancer types, pancreatic ductal adenocarcinoma (PDAC) and hepatocellular carcinoma (HCC). Our goal is to provide a unifying interpretation to the observations from PDAC and/or HCC in some publications reviewed recently [41] that are inconsistent with others for DDX5 to act as a cancer biomarker and target by using the gradually established DDX5’s DNA repair function.

To date, PDAC and HCC are the only cancer types where low levels of DDX5 expression are reported to be associated with higher cancer malignancy and poor prognosis. The multifaceted DNA repair function of DDX5 (Figs. 1 and 2) in cancer can explain the inconsistency with other studies which have identified DDX5 as a biomarker of cancer progression and a therapeutic target for cancer treatment [18–20].

#### DDX5 and pancreatic cancer

A study of 230 chemotherapy-untreated PDAC specimens revealed that low DDX5 expression was associated with disease progression, and the overall median survival time was 24 months with low DDX5 versus 38 months with high DDX5 [42]. While this is a surprising result, this may be explained by DDX5 being involved in different situations of DNA repair function in cancer (R-loop resolution, p53-controlled DNA stability and other DNA damage repair, Table 1, Figs. 1 and 2) as reviewed above. It is well known that pancreatic cancer is the most-difficult-to-treat lethal malignancy with high genome DNA instability persistence and genetic heterogeneity among metastatic cells [43, 44]. This high DNA instability seen in PDAC cells would kill themselves if there was a lack of a DNA repair mechanism. In other words, high DDX5 expression will decrease DNA instability by activating the DDX5-mediated DNA repair surveillance mechanism to reduce pancreatic cancer malignancy. Thus, patients with high DDX5 in tumors may see delayed tumor progression, accounting for better prognosis. However, because in addition to DNA repair (Figs. 1 and 2), DDX5 is also involved in various cancer cell survival functions (e.g., immune suppression, cancer glucose and lipid metabolic control etc., see review below), and targeting DDX5 to inhibit its expression and/or induce its degradation will result in massive DNA damage and apoptosis. Consistent with this notion, after the non-DDX5-targeting drug camptothecin (CPT) treatment, the surviving cancer cells have high DDX5, while the apoptotic cells have low DDX5 [45], indicating that DDX5 is a treatment-resistant factor. Furthermore, the studies from Ling et al. found that targeting DDX5 by the small molecule FL118 exhibits high efficacy to inhibit PDAC patient-derived xenograft (PDX) tumor growth and induces tumor regression/elimination in immunocompromised mice (see below for more information) [46].

#### DDX5 and liver cancer

The role of DDX5 in liver cancer was discussed in our 2021 review [19]. Briefly, the observations include: (i) the mRNA expression of Dgcr8, p68/DDX5, p72, Dicer, Ago3, Ago4 and Piwil4 is significantly decreased in human primary HCC compared to the non-cancerous liver, and Dicer and p68 (mRNA) reduction in HCC is associated with poor prognosis [47]. (ii) HCC patient’s tumors with chronically infected hepatitis B virus (HBV) express reduced DDX5 with poor prognosis [48]. DDX5 KD (DDX^KD^) in HBV-infected hepatocytes increases HBV replication, drug resistance and Wnt signaling, while restoration of DDX5 suppresses HBV and Wnt signaling [49]. DDX5 promotes Stat1 mRNA translation and in turn, Stat1 mediates the IFN response in HCC cells and liver tumors, and DDX5^KD^ reduced both Stat1 and the antiviral effect of IFN-α on HBV replication [50]. (iii) DDX5 promotes autophagy and suppresses liver tumorigenesis, and patients with low DDX5 expression showed poor prognosis [51].

One possibility for such observations (in the liver cancer reviewed above) that contradicts the oncogenic role of DDX5 in other cancer types could be that DDX5 has high genome DNA repair capacity in HCC to control HCC malignancy in the defined conditions. In other words, the observations may be similar to the PDAC case discussed above [42]. PDAC is well known to have a dense extracellular matrix (ECM) called “desmoplasia” which is a barrier for drug delivery. HCC has a similar complex ECM [52] and observations from recent studies suggest a role of proteoglycan Agrin formulating a corrupt ECM network [53, 54]. While the functional interplay between DDX5 and Agrin for oncogenesis and treatment resistance needs to be dissected, HCC may present excessive constitutive DNA instability. Low DDX5 expression may facilitate genome instability-mediated HCC malignancy by decreasing DNA damage repair surveillance. In other words, the DNA repair function of DDX5 could be expected to tilt the HCC aggressiveness balance similar to PDAC.

Another possibility for the role of DDX5 observed in HCC which is not fully consistent with other cancer types could be the DDX5-mediated overall gene expression-collective outcome. For example, in regard to DDX5 acting as a transcription co-activator to upregulate the expression of different genes in the case of DDX5 in resolving G-quadruplexes (G4) structure for transcription, Wu et al. reported that DDX5 is extremely proficient at unfolding a DNA G4 structure in the Myc proximal promoter region (MycG4 that functions as a transcriptional silencer) to turn on the Myc oncogene expression in breast and prostate cancer [55]. Song et al. reported that Dbp2 (the yeast DDX5) binds MycG4 with a high affinity to resolve the MycG4 structure and to have stronger MycG4 folding-promoting activity than DDX5 [56]. Diffendall et al. reported that in parasites, Plasmodium falciparum (Pf), Pf-DDX5 interacts with the non-coding RNA (ncRNA) RUF6 in the RUF6 protein complex that interacts with RNA Pol II to promote the expression of the human host-interacting var gene of Pf for progression, which is likely through resolving the G4 structures in the var gene [57]. In contrast, Sun et al. reported that DDX5 facilitate Stat1 mRNA translation by binding to and resolving the G4 structure at the Stat1 mRNA 5'UTR and in turn, Stat1 mediates the IFN response in HCC cells and liver tumors [50]. DDX5^KD^ reduced both Stat1 and the antiviral effect of IFN-α on HBV replication [50].

Nevertheless, virus infection into HCC cells may further contribute to genomic instability [58], which may make HCC more complex and different from HCC without virus infection. In this regard, studies also revealed that DDX5 is upregulated in HCC, and silencing of the terminal differentiation-induced ncRNA (TINCR) inactivates AKT signaling, which is rescued by DDX5 overexpression [59]. Consistently, DDX5^KD^ decreased Akt as well as p-Akt (S473) expressions [60]. These findings suggested that DDX5 facilitated HCC cell growth via the Akt signaling pathway. The authors therefore concluded that DDX5 played a crucial role in HCC proliferation and tumorigenesis and may be a novel prognostic marker and potential therapeutic target for HCC [60]. Additionally, it was also shown that HSP90 directly interacts with DDX5 to prevent DDX5 degradation, and the accumulated DDX5 induces HCC malignancy [61]. Furthermore, DDX5 silencing blocked in vivo tumor growth in a murine HCC xenograft model; and high levels of HSP90 and DDX5 were associated with poor prognosis indicating a potential therapeutic biomarker target for HCC [61].

Together, while we may beg the question of why DDX5 can delay or inhibit HCC development and malignancy, the role of DDX5 in HCC with, versus without, virus infection may involve overlapped but distinct intracellular events, extracellular events, and innate immunity events in the tumor microenvironment (TME). Nevertheless, further investigation of the role of DDX5 in such complex TME in HCC with, versus without, virus infection may unravel additional unexpected roles of DDX5 as a biomarker and target in oncogenesis.

### DDX5 in immune suppression

**Fig. 3:**
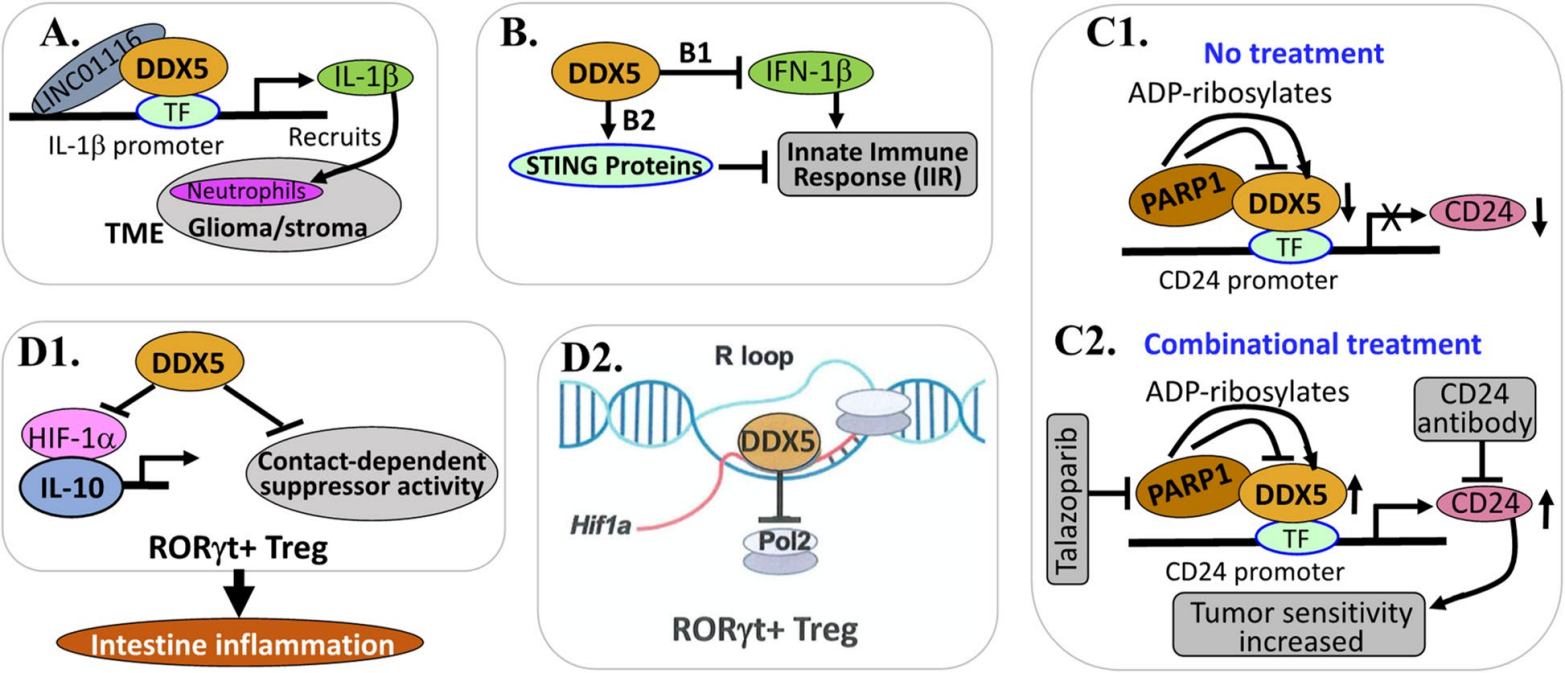
DDX5 functionally suppresses immune system: **A** DDX5 is involved in transcriptional expression of IL-1beta to recruit neutrophils to glioma. **B** DDX5 stabilizes STING protein and blocks IFN-beta production to inhibit innate immune responses (IIRs). **C** PARP1 binds to and ADP-ribosylates DDX5 to inhibit DDX5 and promote CD24 expression for immune suppression. **D1** DDX5 inhibits HIF1α-mediated IL-10 expression and contact-dependent suppressor function in RORγt^+^ T_reg_ and promotes T cell–mediated inflammation in the intestine. **D2** DDX5 resolves the R-loop to block RNA Pol II loading and inhibit *Hif1α* transcription

This is an emerging new area for DDX5 to be involved in the suppression of immune system. We summarized several recent studies in Fig. 3, which are reviewed in a more detail below.

Tumor-associated neutrophils (TANs) promote tumor progression, invasion and metastasis through crosstalk with growth factors, chemokines, inflammatory factors, and other immune cells in TME and are gradually being recognized as a cancer treatment target [62, 63]. Currently, the underlying mechanism of TANs being recruited to glioma remains unknown. In this regard, the study from Wang et al. revealed that the expression of the lncRNA LINC01116 is significantly upregulated in glioma, and positively associated with clinical malignancy and poor survival in glioma patients [64]. Mechanistically, LINC01116 directly binds to and recruits DDX5 to the IL-1β gene promoter in glioma cells to increase the expression of IL-1β which in turn promotes glioma progression and recruits neutrophil to glioma (Fig. 3A) [64]. Thus, this study provides an example that DDX5 can be involved in immune suppression and cancer progression by directly promoting the production of immune cell suppressing cytokines and then attracting neutrophils into TME.

The studies from Dixon et al. revealed that SYNCRIP, MEN1, DDX5, snRNP70, RPS27a, and AATF are the Stimulator of Interferon Genes (STING) partners and are novel modulators of dsDNA-triggered innate immune responses (IIRs) [65]. The authors found that in contrast to siRNA KD of SYNCRIP, MEN1 or SNRNP70 not affecting STING protein levels, siRNA KD of DDX5 caused a reduction in STING protein levels (Fig. 3B) [65], suggesting that DDX5 is required for STING stability. Furthermore, in contract to that the siRNA KD of SYNCRIP, MEN1 or snRNP70 all reduced IFN-β production by more than 50%, it was surprisingly observed that siRNA KD of DDX5 significantly enhanced IFN-β induction from both DNA and poly(I:C) immune stimulation (Fig. 3B) [65], indicating that DDX5 plays an inhibitory role in IFN-β production, which is opposed to other STING partners in the regulation of IFN-β production. This observation is especially intriguing given that DDX5 siRNA treatment caused a reduction in STING protein levels and suggests that DDX5 functions as a negative regulator of both DNA- and RNA-triggered IIRs [65]. Collectively, their studies indicated that SYNCRIP, MEN1, and SNRNP70 are positive regulators of dsDNA-stimulated IIRs, while DDX5 is a negative regulator of dsDNA- and dsRNA-stimulated IIRs. In other words, DDX5 can inhibit IIRs. Therefore, pharmaceutical inhibition or degradation of DDX5 such as by a small molecule for cancer treatment would at the same time stimulate IIR activation to further help cancer treatment.

One role of the immunosuppressive factor (checkpoint), CD24 is to act as a glycosylated small protein on the immune and cancer cell surface against phagocytosis. However, the role of IIRs in targeted anti-PARP1 therapy remains poorly understood. In this regard, the studies from Chen et al. found that PARP1 suppresses the transcription of CD24 in pancreatic cancer cells; and targeting CD24 by a CD24 locking mAb increased the phagocytosis of pancreatic cancer cells by macrophages [66]. Mechanistically, PARP1-mediated inhibition of CD24 transcription was mediated by DDX5. PARP1 binds to, and inhibits DDX5 by ADP-ribosylating DDX5 in pancreatic cancer cells (Fig. 3C1), which can be abolished by PARP1 inhibitor, talazoparib, while PARP1 inhibition increased DDX5 expression and binding to the CD24 promoter [66]. Consistently, high DDX5 expression in pancreatic cancer tissues was correlated to high CD24 expression, while the PARylation activity of PARP1 was inversely correlated with CD24 expression [66]. Furthermore, co-targeting PARP1 (with talazoparib) and CD24 (with anti-CD24 mAb) elicited synergistic antitumor effects in human pancreatic cancer animal models (Fig. 3C2) [66]. Thus, the study suggests that DDX5 is involved in immune checkpoint molecule CD24-mediated resistance in pancreatic cancer. This is important because innate immunity in tumor cell surveillance and eradication has been increasingly recognized for its role in the modulation of anti-tumor immunity [67].

Ma et al. reported that DDX5 is a negative regulator of the retinoic acid-receptor (RAR)-related orphan receptor γ t (RORγt)-expressing regulatory T cells (RORγt + T_regs_) suppressor activities [68], and that loss of DDX5 unleashes IL-10 production potential and suppressor activity in RORγt + T_reg_ and protects against weight loss and pathology in murine models of T cell–mediated intestinal inflammation (Fig. 3D1) [68]. Specifically, these authors found that (i) hypoxia-induced factor 1α (HIF1α) is the master TF for IL-10 expression in RORγt + Tregs; (ii) DDX5 restricts the expression of HIF1α by promoting R-loop disassembly and restricting RNA Pol II recruitment on the HIF1α gene locus in RORγt + T_regs_ (Fig. 3D2). Thus, HIF1α transcription decreases and in turn, decreasing HIF1α downstream target IL-10 gene expression in RORγt + Tregs (Fig. 3D1, D2); and (iii) T cell-specific DDX5 KO (DDX5^ΔT^) mice augment RORγt + T_reg_ suppressor activities and are better protected from intestinal inflammation [68]. Consistently, inhibition of IL-10 signaling or genetic ablation or pharmacologic inhibition of HIF1α restores enteropathy susceptibility in DDX5^ΔT^ mice [68]. This means that the inhibition of DDX5 would resist enteropathy, while sensitizing cancer cell death. Given that the DDX5-HIF1α-IL-10 pathway is conserved in mice and humans [68], pharmacologic inhibition of DDX5 can activate the HIF1α–IL-10 pathway in both mouse and human T cells. Thus, DDX5 provides a potential therapeutic target for intestinal inflammatory diseases and the treatment of cancers. Interestingly, this is an example for DDX5 to act as a transcription co-repressor (in immune Treg cells) but not a transcription co-activator (in cancer cells). Accumulated evidence indicates that DDX5 could select distinct downstream targets based on the organ/cell types and/or regulation context. For example, in colon, DDX5 controls complement component 3 (C3) expression to promote tumorigenesis [69]. In contrast, in the small intestine, DDX5 controls fatty acid-binding protein 1 (FABP1) expression to promote tumorigenesis [69]. Such a feature would make DDX5 an ideal target for treating human cancer in terms of achieving high efficacy with low toxicity (see more detailed review on this paper later).

Additionally, it is possible that the clinical outcomes through targeting DDX5 for cancer treatment could be better than the antitumor efficacy obtained in the preclinical studies using immune-deficient mice. This is because all cancer patients have their immune system intact and targeting DDX5 for the treatment of cancer could eliminate the DDX5-mediated immune suppression in patients to further help eliminate tumors.

### DDX5 in cancer metabolic control

**Fig. 4:**
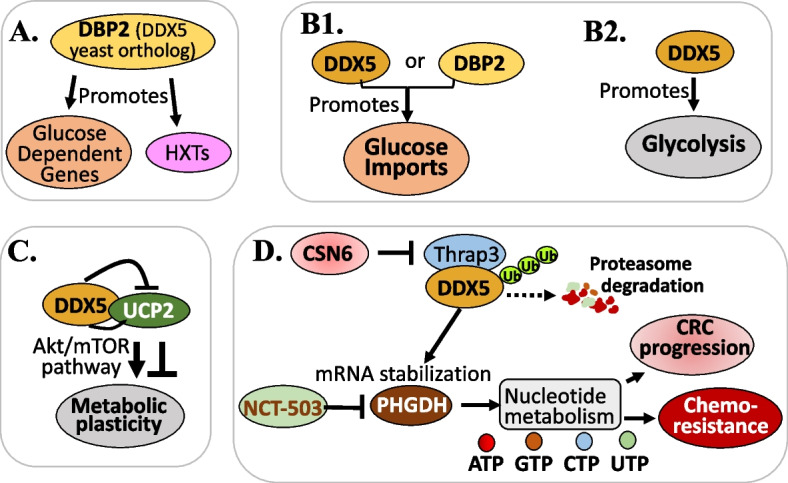
DDX5 plays a role in cancer metabolic control: **A** DBP2, a DDX5 yeast ortholog, promotes glucose-dependent gene expression and upregulates the expression levels of HXTs. **B1** DDX5 or DBP2 is required for efficient glucose import. **B2** DDX5 promotes glycolysis. **C** DDX5 recruiting with UCP2 at least partially contributes to the metabolic plasticity of NSCLCs via the AKT/mTOR pathway. **D** CSN6-mediated induction of PHGDH and metabolic reprogramming relies on DDX5, specifically, the E3 ligase β-Trcp interacts with, ubiquitinates and degrades DDX5, which can be blocked by CSN6 to stabilize DDX5 protein and in turn promote DDX5-mediated PHGDH mRNA stabilization, leading to metabolic reprogramming in CRC cells thus, promoting tumorigenesis reflected by poor CRC patient prognosis

This is a very interesting area for DDX5 to regulate cancer metabolism. We summarized relevant studies in Fig. 4, which are reviewed in a more detail below.

Beck et al. found that DBP2 (yeast ortholog of DDX5) promotes glucose-dependent gene expression and upregulates the levels of transcripts of hexose transporters (HXTs) (Fig. 4A) [70], which are known to be the rate-limiting step in sugar catabolism and provide the sole portal for the cellular import of fructose, mannose, and glucose in yeast [71, 72]. This finding suggests that DBP2 is a key integrator of sugar catabolism and glycolysis for energy homeostasis. Furthermore, Xing et al. from the same group also performed a comparative study between human DDX5 and yeast DBP2, and found that (i) human DDX5 possesses both ~ 10-fold higher unwinding activity and higher RNA-binding affinity than DBP2, which is in part due to the presence of a carbos-terminal extension (CTE) domain in DDX5 [73]; (ii) ectopic expression of DDX5 complements cell growth and transcriptional fidelity defects in S. cerevisiae yeast cells without the DBP2 gene (dbp2Δ), suggesting functional conservation [73]; (iii) DBP2 and DDX5 are required for the efficient import of glucose in yeast and mouse AML12 hepatocyte cell models (Fig. 4B1), respectively, and dbp2Δ yeast cells show HXT gene mis-regulation [73]. Furthermore, the expression of DDX5 or DDX5^ΔCTE^ not only rescued dbp2Δ cells’ import defects, but also stimulated ∼50% increase of the glucose marker/surrogate import compared with wild-type cells [73]; and (iv) AML12 cells with DDX5^KD^ exhibit both basal and maximal glycolysis rate decrease with respiration (oxygen consumption rates) increase due to reduced glycolytic activity, indicating the promotion of glycolysis by DDX5 (Fig. 4B2), whereas non-glycolytic acidification is not affected [73]. Based on this study and given that the GLUT genes are upregulated in cancers [74] and GLUT inhibitors are currently in clinical trials in different regimens [75], we can safely anticipate that DDX5 will regulate the glucose transporters (GLUTs) similarly in mammalian cells. However, another study from Xing et al. (2020) reported that (i) DDX5 is overexpressed in small cell lung cancer (SCLC) cell lines and is required for SCLC cell growth [76]. DDX5 depletion in SCLC cells downregulates genes involved in oxidative phosphorylation and impaired oxygen consumption which reduced the TCA cycle intermediate succinate [76], suggesting that DDX5 is also required for mitochondrial energy metabolic function. Still, the authors concluded that the oncogenic role of DDX5, at least in part, manifests as upregulation of respiration, supporting the energy demands of cancer cells [76].

We should point out that DDX5 is involved in glucose metabolism would impact on human disease treatment including, but not limited to cancer. It is known that cancer cells acquire glucose dependence for aerobic glycolysis (Warburg effect) to fulfill the need for massive macromolecule synthesis of fast-growing cancer cells with reduced mitochondria-dependent apoptosis [77]. This would provide a novel opportunity for targeting DDX5 to disrupt cancer metabolism. Consistent with this notion, Mazurek et al. found that silencing DDX5 in acute myeloid leukemia (AML) cells induces glycolysis dysregulation, elevates ROS production and induces apoptosis by downregulating the expression of glucose metabolism-relevant genes [78].

Additionally, it is known that mitochondrial uncoupling protein 2 (UCP2) is implicated in physiological and pathological processes related to glucose and lipid metabolism, and acts as a glucose level modulator through signaling pathways [79]. In this regard, it was recently demonstrated by Cheng et al. that UCP2 expression in melanoma is associated with elevated T cell infiltration in patient melanoma tumors and prolonged patient survival rates, and is associated with antitumor immune states in TME as well as conventional type 1 dendritic cells (cDC1) and CD8 + T cell infiltration in tumors [80, 81]. Consistently, UCP2 induction sensitizes melanomas to PD-1 blockade treatment and elicits effective antitumor responses [80, 81]. Mechanistically, UCP2 reprogrammed the immune state of the TME by altering its cytokine milieu in an interferon regulatory factor 5 (IRF5)-dependent manner [80, 81]. In this regard, Yang et al. reported that both the computational model docking and experiments showed that DDX5 interacts with UCP2 in H1299 non-small cell lung cancer (NSCLC) cells, and the recruiting of DDX5 with UCP2 at least partially contributes to the metabolic plasticity of NSCLCs via the AKT/mTOR pathway (Fig. 4C) [82]. Given that their data showed that there is no correlation of DDX5 and UCP2 expression [82], as well as the early reviewed immune-suppression role of DDX5 [64–66, 68], DDX5 likely acts as a UCP2-independent regulator to negate UCP2 functions (Fig. 4C), which provides an additional way for DDX5 to elicit its immune suppression function, and also to play a modulatory role in glucose metabolism.

Additionally, it is known that the constitutive photomorphogenesis 9 (COP9) signalosome (CSN) consists of eight subunits (CSN1 to CSN8) in mammalian cells. Among the CSN subunits, CSN5 and CSN6 are the only two that each contain an Mpr1p and Pad1p N-terminal (MPN) domain and are implicated in ubiquitin-mediated proteolysis of important mediators in carcinogenesis and cancer progression [83, 84]. In this regard, Zou et al. reported that CSN6 mediates nucleotide metabolism to promote tumor development and chemoresistance in colorectal cancer (CRC) [85]. Specifically, the authors found that CSN6 is involved in promoting purine, pyrimidine and nucleotide synthesis through increasing the expression of phosphoglycerate dehydrogenase (PHGDH) [85], a key enzyme in the de novo serine synthesis pathway for nucleotide metabolism [86]. However, CSN6-mediated induction of PHGDH and metabolic reprogramming relies on DDX5 [85]. Their studies revealed that the E3 ligase β-Trcp interacts with and degrades DDX5, which could be blocked by CSN6 via inhibiting ubiquitin–proteasome-mediated protein degradation to stabilize DDX5 protein and in turn promote DDX5-mediated PHGDH mRNA stabilization, leading to metabolic reprogramming in CRC cells and in turn, the CSN6-DDX5-PHGDH axis promotes tumorigenesis and is associated with poor CRC patient prognosis (Fig. 4D) [85]. Furthermore, the authors demonstrated that butyrate, as a potential CSN6 antagonist, in combination with 5-FU shows increased antitumor efficacy [85].

Based on the updated publications in the literature reviewed above, we can conclude that DDX5 is involved in promoting the reprogramming of the metabolism of glucose, lipid, and nucleotide in cancer. Thus, pharmacological inhibition or degradation of DDX5 would also block the abnormal reprogramming of glucose (Warburg effect), lipid and nucleotide metabolisms in cancer to induce apoptosis and cancer cell killing.

### DDX5 in virus infection and replication promotion and inhibition

**Fig. 5:**
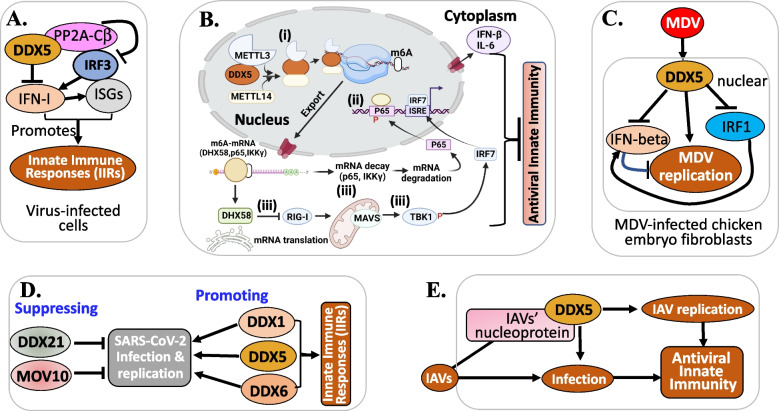
DDX5 modulates virus infection and replication: **A** DDX5 suppresses IFN-I antiviral IIRs by interacting with PP2A-Cβ to deactivate IRF3 to inhibit IFN-I production. **B** DDX5 inhibits antiviral innate immunity by promoting m6A-methylated antiviral transcripts. (i) DDX5 interacts with METTL3 to regulate methylation of mRNA through affecting the METTL3-METTL14 heterodimer complex; (ii) DDX5 promotes m6A modification and nuclear export of DHX58, p65, and IKKγ transcripts by binding the conserved UGCUGCAG element; (iii) stable IKKγ and p65 transcripts underwent YTHDF2-dependent mRNA decay, whereas DHX58 translation was promoted, resulting in the inhibited antiviral IIRs by DDX5 blocking the p65 pathway and activating the DHX58-TBK1 pathway. As a result, DDX5 suppresses antiviral innate immunity. **C** DDX5 suppressed IFN-β production and inhibited the expression of IRF1 and thus, promoted MDV replication. **D** DDX1, DDX5 and DDX6 promoted SARS-CoV-2 infection and replication by suppressing host IIRs, while DDX21 and MOV10 suppressed SARS-CoV-2 infection and replication. **E** DDX5 suppresses antiviral innate immunity and promotes replication of IAV

The oncogenic potential of many viruses is well characterized [87, 88]. Additionally, the significance of DDX5 in promoting virus infection and replication has been identified. Therefore, virus infection-associated cancer may be one of the DDX5’s oncogenesis mechanisms. Bonaventure and Goujon recently provided an overview of the DExH/D-box helicases at the frontline of intrinsic and innate immunity against viral infections [89]. As reviewed, DExH/D-box helicases play multiple roles in viral life cycles. Some act as viral sensors (DDX3, DDX41, DHX9, DDX1/DDX21/DHX36 complex), and others have roles in innate immune activation (DDX60, DDX60L, DDX23), and still others (DDX39A, DDX46, DDX5 and DDX24) act as negative regulators and impede interferon (IFN) production upon viral infection [89]. Furthermore, studies indicated that DDX56, DDX17 (a paralog of DDX5), DDX42 intrinsically restrict viral replication [89]. Here, we also cite two DDX5-focused review articles for those who want to review the previous studies on DDX5 with viral infection and innate immunity reaction [90, 91]. While DExH/D-box helicases could either promote or inhibit the viral infection, evidence indicated that DDX5 can act as a helper or inhibitor for viral infection and play a role in innate immune suppression [91]. In this section, we review relevant new publications on the role of DDX5 in viral infection and innate immune responses (IIRs). We summarized the studies in Fig. 5 from the updated publications, which are reviewed in a more detail below. After review, we conclude that DDX5 is an antiviral target and initiates innate immune suppression by restricting the production of immune-stimulatory IFNs and/or cytokines. Therefore, pharmacological inhibition or degradation of DDX5 may provide effective strategies to enhance IIRs against virus infection, replication, and other human diseases including cancer. Detailed review is provided below.

Zan et al. reported that DDX5 suppresses type I interferon (IFN-I) antiviral IIRs [92]. Specifically, these authors found that (i) DDX5 is a negative regulator of IFN-I production in antiviral responses (Fig. 5A); (ii) DDX5 KD significantly promotes DNA or RNA virus infection-induced IFN-I production and IFN-stimulated genes (ISGs) expression, and renders the mice more resistant to viral infection and enhanced antiviral innate immunity, while ectopic expression of DDX5 inhibited IFN-I production and promoted viral replication; and (iii) mechanistically, DDX5 specifically interacts with serine/threonine-protein phosphatase 2 A catalytic subunit β (PP2A-Cβ), which can be enhanced by viral infection (Fig. 5A) [92]. Furthermore, PP2A-Cβ interacted with and deactivated IFN regulatory factor 3 (IRF3) to inhibit IFN-I production (Fig. 5A) [92]. In short, the data from Zan et al. support that DDX5 suppresses IFN-I antiviral IIRs by interacting with PP2A-Cβ to deactivate IRF3 (Fig. 5A) [92]. In our view, this study has provided new perspectives for clinical application of DDX5 to treat cancer, viral infection, and other human diseases, because DDX5-mediated inhibition of IFN-I signaling in IIRs grants DDX5 an advantage as a target not only for disease treatment but also for activating patients’ IIRs against the disease during treatment.

Xu et al. reported that DDX5 promotes viral infection via regulating N6-methyladenosine (m6A) levels on the DHX58 and NF-κB transcripts to dampen antiviral innate immunity [93]. They found that (i) DDX5 interacts with the m6A writer METTL3 (methyltransferase 3) to regulate methylation of mRNA through affecting the METTL3-METTL14 heterodimer complex (Fig. 5B); (ii) DDX5 promotes m6A modification and nuclear export of DHX58, p65, and IKKγ transcripts by binding the conserved UGCUGCAG element in IIRs after viral infection (Fig. 5B); (iii) stable IKKγ and p65 transcripts underwent YTHDF2-dependent mRNA decay, whereas DHX58 translation was promoted, resulting in the inhibited antiviral IIRs by DDX5 via blocking the p65 pathway and activating the DHX58-TBK1 pathway after infection with RNA virus (Fig. 5B) [93]. As a result, DDX5 suppresses antiviral innate immunity (Fig. 5B). These authors concluded that DDX5 serves as a negative regulator of innate immunity by promoting RNA methylation of antiviral transcripts and consequently facilitating viral propagation [93]. In this regard, DDX5 would be a good target for the restriction of virus infection and replication as well as activating IIRs.

Another study from Xu et al. found that DDX5 is hijacked by an avian oncogenic herpesvirus to inhibit IFN-β production and promote viral replication [94]. These authors showed that (i) Marek's disease virus (MDV), an avian oncogenic herpesvirus, inhibits the production of IFN-β through increasing the expression and nuclear aggregation of DDX5 which in turn inhibits the expression of IFN regulatory factor 1 (IRF1) in chicken embryo fibroblasts (CEFs) (Fig. 5C); and (ii) MDV replication is suppressed, and the production of IFN-β is promoted in the DDX5 silencing CEFs [94]. These observations indicate that in non-human, DDX5 also plays a role in innate immunity by suppression of IFN production to facilitate viral infection and replication (Fig. 5C). Thus, pharmaceutical inhibition or degradation of DDX5 would enhance IIRs against virus invasion and replication. Additionally, the study from Ariumi revealed that while RNA helicases DDX21 and MOV10 suppress SARS-CoV-2 infection and replication, DDX1, DDX5 and DDX6 are required for SARS-CoV-2 infection and replication by suppressing host IIRs (Fig. 5D) [95]. Furthermore, the study from Zhao et al. found that (i) DDX5 promotes replication of influenza A virus (IAV) in lung cancer A549 cells by its N terminus to interact with IAV’s nucleoprotein (independent of RNA) (Fig. 5E); and (ii) DDX5 suppresses antiviral innate immunity induced by IAV infection (Fig. 5E). Mechanistically, DDX5 downregulated the mRNA levels (mRNA decay) of IFN-β, IL-6, and DHX58 via the METTL3-METTL14/YTHDF2 axis against the innate immune system as portraited in Fig. 5B [96].

Together, these studies indicate that DDX5 promotes virus infection and replication and suppresses IIRs. Thus, targeting DDX5 would counteract virus infection and replication and enhance IIRs against human disease including virus infection and cancer.

### DDX5 in inflammation and negative impacts of microbiota in intestines

**Fig. 6:**
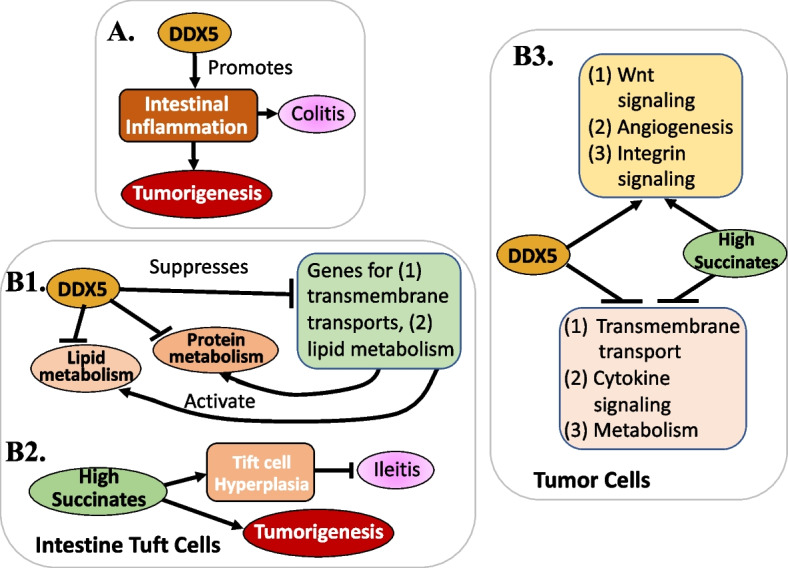
**A** DDX5 is involved in intestinal inflammation to course colitis and tumorigenesis: **B1** DDX5 inhibits lipid and protein metabolism in intestine tuft cells by blocking the expression of genes involved in transmembrane transport and lipid metabolism. **B2** High succinate results in tuft cell hyperplasia, which leads to ileitis as well as tumorigenesis. **B3** DDX5 promotes Wnt signaling, angiogenesis and integrin signaling, while suppressing transmembrane transport, cytokine signaling and metabolism in cancer cells and high succinate could mimic DDX5’s such effects and roles, which will need further investigation

Altered human gut microbiomes can impact the long process of CRC development in many ways. This includes, but may not be limited to, induction of host gene mutations, augmentation of host oncogenic signaling cascades, induction of host inflammation, promotion of host immune evasion, co-metabolism of host and dietary components, and aberrant interactions with host genetics and/or epigenetics [97]. On the other hand, probiotics could act as anticancer agents, suppress inflammation, elicit antitumor surveillance and reverse gut microbiota dysbiosis [97]. Therefore, targeted modulation of gut and tumor microbiota through various strategies for cancer patients (e.g., application of antibiotics, probiotics, prebiotics, dietary modulation and fecal microbiota transplantation) could be a helpful part of various cancer therapies which would provide better treatment outcomes during various cancer therapies [98–100]. However, whether DDX5 could affect the function of intracellular and extracellular events that are associated with gut and tumor microbiota-elicited functions has not been recognized in various microbiota-related studies. Here, we review this specialized new area to extend the vision on DDX5’s role and augment the potential of cancer therapeutics using DDX5 as a biomarker and target. We summarized the studies in Fig. 6, which are reviewed in a more detail below.

By comparing the RNA profiles of colonic intestine epithelial cells (IECs) isolated from wild type IEC (WT^IEC^) mice versus from DDX5-KO-IEC (DDX5^ΔIEC^) mice, Abbasi et al. found that DDX5-dependent RNA programs of the colonic IECs were enriched with genes involved in immune response activation [69]. This suggests that DDX5 may be involved in colonic IECs’ negative/harmful inflammation response. In a dextran sodium sulfate (DSS)-induced colitis model, DDX5^ΔIEC^ animals experienced less weight loss and recovered faster than their WT^IEC^ cohoused littermates by day 9 [69]. This observation further strengthens the notion that pharmaceutical inhibition or degradation of DDX5 would not only induce little toxicity to normal tissues but may even help animals avoid disease like colitis shown here. More supportive observations from their studies indicated that colons from DSS-challenged DDX5^ΔIEC^ animals showed milder histological pathology, particularly in matrices scoring for immune infiltration, submucosal inflammation, and abnormal crypt density [69]. More importantly, colonic tissues from ulcerative colitis (UC) patients have higher DDX5 expression than healthy controls [69], similar to those reported previously [101]; and DDX5 reduction positively correlates with UC patients responding favorably to anti-TNF therapy [69]. Together, DDX5 abnormally regulates the epithelial immune response program and contributes to unfavorable inflammation reactions for disease seriousness in the colon (Fig. 6A). Consistent with this study, the study from Zheng et al. demonstrated that Nogo-B interacts with DDX5/p68 to control miR-155 maturation, and Nogo deficiency significantly reduced DSS-induced weight loss, colon length and weight reduction, and inflammatory cells accumulation in the intestinal villus, and blocking p68/DDX5 inhibited the expression of Nogo-B, miR-155, TNFα, IL-1β and IL-6 [102], which are known to be involved in disease inflammation. Thus, the fact that different proteins use DDX5 to execute its effects on and being involved in disease seriousness would make DDX5 an ideal target for disease treatment.

Similar to the finding described above from Abbasi et al. [69], another recent report showed that activation of the tuft cell is also linked to intestinal inflammation and provides protection against T-lymphocyte-mediated ileitis on anti-CD3ε challenge without knowing if this is relevant to DDX5 [103]. However, by using the transcriptomes of steady-state IECs derived from two matched pairs of WT^IEC^ and DDX5^△IEC^ male littermates available from their previous study [69], Long et al. performed a lineage-specific GSEA analysis. The analysis indicated that DDX5 may have a unique role in regulating tuft cell differentiation and/or function in the intestine [104]. With this encouraging finding, these authors further assessed the role of DDX5 in tuft cell specification and function in control (WT^IEC^) versus DDX5^△IEC^ mice using transcriptomic approaches [104]. They found that DDX5^△IEC^ mice harbored a loss of intestinal tuft cell populations, modified microbial repertoire, and decreased susceptibilities to ileal inflammation and colonic tumorigenesis [104]. Specifically, the studies demonstrated that DDX5 negatively regulates tuft cell lipid and protein metabolic programs (Fig. 6B1) [104]. Importantly, despite a significant loss of tuft cells in the DDX5^△IEC^ intestine, a small population of tuft cells can be generated in the absence of DDX5 and DDX5 showed no influence on tuft cell morphology [104]. Furthermore, the authors found that ten of the genes involved in transmembrane transport and lipid metabolism that were upregulated in DDX5^△IEC^ tuft cells were direct targets of DDX5 (Fig. 6B1) [104]. This reveals a novel role of DDX5 as a repressor of transmembrane transport and lipid metabolic programs in tuft cells of the small intestine (Fig. 6B1) and thus, extends the role of DDX5 in modulating the microbial community in the intestine. Convincingly, these authors further demonstrated that succinate (a microbial-derived metabolite)-induced tuft cell hyperplasia protects against ileitis and restores colon tumorigenic potential in DDX5^△IEC^ mice (Fig. 6B2) [104]. Long et al. further tested whether the tuft cell numbers reduced in the small intestine of DDX5^ΔIEC^ mice may result in enhanced susceptibility to ileitis [104]. The authors found that 50% of the DDX5^ΔIEC^ mice challenged in the model succumbed to the disease by day 18 [104]. Of those that survived, mononuclear immune cells from their ileal lamina propria had elevated transcripts encoding the inflammatory cytokine TNF [104]. Furthermore, transcriptomic analysis of control and DDX5 deficient tumors revealed that the succinate-treated tumor-susceptible background APC^ΔIEC^DDX5^ΔIEC^ mice had higher colonic tumor counts than those treated with vehicle [104]. Thus, succinate at least partially rescues DDX5 deletion-induced tumor formation inhibition (Fig. 6B2). In tumor cells, DDX5 promotes Wnt signaling, angiogenesis and integrin signaling, while suppressing transmembrane transport, cytokine signaling and metabolism (Fig. 6B3) [104]. Thus, high succinate may mimic DDX5’s such effects and roles (Fig. 6B3), which will need further investigation.

In our view, these observations provide a potential that downregulation of DDX5 in the intestine may improve microbial repertoire to benefit health. In other words, based on this case, pharmaceutical inhibition/degradation of DDX5 for cancer therapeutics would not only eliminate cancer but may also benefit patients through improving cancer patients’ intestine microbiota. Consistently, the study from Kandeel et al. (2023) reported that Memantine and Augmentin could increase spatial memory in healthy rats, and improved spatial memory in Alzheimer's disease (AD) rats, which linked to the expression of DDX5 decrease in the AD-treated groups [105]. This intriguing observation provides a possibility that DDX5 decrease may benefit both healthy and AD rats by improving spatial memory.

Together, these results highlight the critical roles of epithelial DDX5 in protecting against ileal inflammation yet contributing to colonic tumorigenesis [104]. However, this seemingly paradoxical function of DDX5 provides the ideal situation when targeting DDX5 for cancer therapeutics as it provides the ability to potentially avoid adverse side effects on physiology and normal tissues while causing cancer cell death.

Additionally, consistent with the observation reviewed above [103, 104], other studies from the literature indicate that various situations could drive the extracellular increase of succinate (secreted by cancer cells), which plays a driver role in epithelial mesenchymal transition (EMT) and cancer metastasis [106]. Given that abnormal increases in succinate also enhance the risk of immune disorders linked to diseases like inflammation and cancer [107]. Therefore, an intriguing research area is whether DDX5 is involved in abnormal increases of succinate or DDX5 and succinate are independent in-parallel molecule signals.

Mechanistically, DDX5 promotes CDC42 protein synthesis through a post-transcriptional mechanism to license tuft cell specification [104]. However, the DDX5-CDC42 axis is dispensable for tuft cell hyperplasia in response to IL-13 [104]. This is important because the DDX5-CDC42 axis is in parallel with, but distinct from the known IL-13 circuit implicated in tuft cell hyperplasia, and both pathways augment the tuft cell commitment factor, *Pou2f3* expression in secretory lineage progenitors [104]. In mature tuft cells, DDX5 not only promotes integrin signaling and microbial responses, but it also represses gene programs involved in membrane transport and lipid metabolism as indicated early [104]. Thus, all these mechanistic studies lay a foundation to understand and support why targeting DDX5 for cancer treatment may induce minute toxicity to issues, while eliminating cancer for patients.

Additionally, it is worthy of mentioning that by comparison with the secretory lineage progenitors, Long et al. found that DDX5 has a limited transcription footprint on intestinal stem cells (ISCs) [104]. Unlike those observed in cells from the CDC42^ΔIEC^ mice [108], Long et al. did not find abnormalities in growth and survival in the DDX5^ΔIEC^ crypts containing ISCs and progenitors [104]. The authors therefore proposed that this likely suggests that the remaining DDX5-modulated CDC42 levels in the DDX5^ΔIEC^ epithelium are sufficient to maintain ISC growth and survival, or alternatively, CDC42 expression in ISCs may be DDX5-independent [104]. In our view in either case, this further indicates that targeting DDX5 for cancer treatment would have low toxicity to normal tissues including ISCs. Nevertheless, based on the results, these authors concluded that DDX5 directs tuft cell specification and function to regulate microbial repertoire and increase disease susceptibility in the intestine [104].

However, in the case of nonalcoholic fatty liver disease and its progressive form, nonalcoholic steatohepatitis (NASH) (two major causes of HCC), Zhang et al. (2022) found that (i) the expression of DDX5 is downregulated in NASH patients, diet-induced NASH mice and NASH-HCC mice [109]; (ii) virus-mediated DDX5 overexpression ameliorates hepatic steatosis and inflammation, whereas its deletion worsens such pathology [109]; (iii) untargeted metabolomics analysis of the mechanism of DDX5 in NASH and NASH-HCC revealed the regulatory effect of DDX5 on lipid metabolism [109]; and (iv) the phytochemical compound hyperforcinol K directly interacted with DDX5 and prevented its ubiquitinated degradation by the E3 ligase TRIM5 [109]. These interesting observations from liver to liver-disease to NASH to HCC are somehow inconsistent with the intestine case reviewed above. However, in consideration of the two unique cancer cases (PDAC, HCC) among all other types of cancer reviewed in the subsection of “DDX5 in general DNA damage repair and cancer malignancy”, the downregulation of DDX5 during liver to lipid-mediated disease progression could be the negative feedback against the disease (i.e., cells trying to slow disease development) and this would need further investigation, from which we may obtain unexpected findings to unify all observations. Nevertheless, one consistent observation is that DDX5 is involved in the regulation of glucose and lipid metabolism as indicated in both studies [104, 109].

Overall, the relevant publications reviewed above suggest that DDX5 could be an ideal cancer therapeutic target to conquer cancer. Targeting DDX5 may not only produce little toxicity to normal tissues but the pharmaceutical inhibition or degradation of DDX5 may also produce positive effects against DDX5-induced inflammation thus benefiting the overall health of cancer patients. These would be great advantages in the application of DDX5 as a target for cancer therapeutics.

### DDX5 as a biomarker and target in cancer initiation, progression, and resistance

There are several review articles that have focused on DDX5 (p68) as a cancer target and biomarker involved in tumorigenesis and cancer development for cancer therapy [18, 19, 110]. They have been cited in order to help readers gaining an overview of the relevant literature. In this section, we will focus on recent key publications relevant to DDX5 acting as a biomarker and target for cancer therapeutics.

Liu et al. performed a comprehensive pan-cancer analysis of the prognostic and immunological roles of DDX5 in human tumors [111]. The analysis revealed that the DDX5 mRNA increase in tumors is related to decreased overall survival (OS), progression-free interval (PFI), and disease-specific survival (DSS) in 3 cancers but increased OS, PFI, and DSS in others [111]. While this is important information, it should be recognized that mRNA may not always reflect the protein situation. As discussed earlier, so far only PDAC and HCC in some publications show that higher DDX5 (protein) is linked to favorable patient outcomes. This could be explained by the function of DDX5 (protein) in DNA damage repair. Additionally, studies indicated that degradation of DDX5 (protein) by FL118 induces cancer cell apoptosis but at the same time induces DDX5 mRNA levels [46]. The other 3 major findings by Liu et al.’s analyses include (i) methylation in the DDX5 promoter is significantly reduced in 8 cancer types [111]; (ii) DDX5 is associated with multiple cellular pathways (e.g., RNA splicing, Notch signaling, and viral carcinogenesis) [111]; and (iii) DDX5 mRNA expression is highly correlated with the infiltration of CD8( +) T cells, cancer-associated fibroblasts (CAFs), and B cells in a wide variety of malignancies [111]. The Li et al.’s last finding in their pan-cancer analysis of DDX5 mRNA appears to be inconsistent with the potential role of DDX5 (protein) in immune suppression. This could be resulted from a low DDX5 (protein)-induced feedback that promotes the synthesis of DDX5 mRNA in tumors (for cell survival), and low DDX5 (protein) in tumor cells may facilitate the infiltration of cancer killing immune cells into TME. On the other hand, CAFs are known to counteract and suppress cancer cell-killing immune cells. In any case, this is an important area for further investigation. With such up-to-date information for DDX5 [111], we found that the following publication is extremely intriguing and worthy of detailed review.

Consistent with the previous observation that intestinal tumorigenesis in Apc^fl/+^Cdx2^Cre+^ mutant (APC^ΔcIEC^) mice is driven by colonic immune cell–mediated inflammation [112], studies from Abbasi et al. revealed that DDX5 was expressed at a significantly higher level in colonic tumors from APC^ΔcIEC^ mice than adjacent normal tissues or IECs isolated from non–tumor-bearing WT Apc mice [69]. Intriguingly, at 4 months of age, APC^ΔcIEC^DDX5^ΔcIEC^ mice had lower incidence of anal prolapse and experienced less weight change compared to APC^ΔcIEC^DDX5^WT^ control mice [69], suggesting that DDX5 KO in normal IECs not only shows no toxicity, but also has a protective role in human disease. Macroscopic tumor numbers in the jejunum, ileum, and colon of the APC^ΔIEC^DDX5^ΔIEC^ mice were significantly lower than those found in the APC^ΔIEC^DDX5^WT^ mice [69], and lesions from the APC^ΔcIEC^DDX5^ΔcIEC^ mice had reduced expression of the Ki67 cell proliferation marker [69]. Together, these observations strongly indicated that epithelial DDX5 promotes colonic tumorigenesis. This observation is clinically relevant because Kaplan–Meier analysis of alive and disease-free survival in two independent cohorts plus the progression-free survival from a third patient cohort, revealed strong associations of the DDX5-associated 20-downregulated gene signature (identified in the colonic IEC RNA-seq study) with worse CRC outcome [69].

**Fig. 7:**
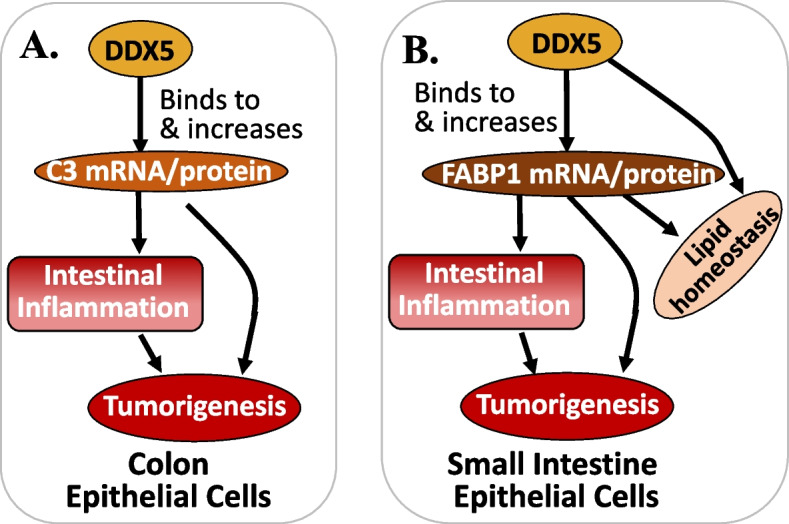
DDX5 promotes inflammation and tumorigenesis in colon and small intestine: **A** DDX5 binds to and promotes C3 mRNA expression, and in turn induces inflammation and tumorigenesis in colon. **B** DDX5 binds to and promotes FABP1 mRNA expression, and in turn (1) induces inflammation and tumorigenesis and (2) plays a role in lipid homeostasis in small intestine

Previous studies indicated that higher expression of complement component 3 (C3) in CRC tumors predicts poor overall and relapse-free patient survival [113, 114]. In this regard, Abbasi et al. found that APC^ΔcIEC^DDX5^ΔcIEC^ mice exhibited a mirrored phenotype of the Apc^mut^C3-deficient mice [69]. Such similarity implies that colonic DDX5 KO in IECs (DDX5^ΔcIEC^) is equivalent to C3-deficiency. Further studies revealed that epithelial DDX5 directly binds C3 mRNA and enhances C3 post-transcriptional expression in colonic IECs (Fig. 7A) [69]. Additionally, among seven significantly altered RNA expression in DDX5-deficient ileal IECs, only the fatty acid-binding protein 1 (FABP1) mRNA-enhanced expression (but not the others) significantly correlates with worse relapse-free survival in CRC patients [69]. Consistent with the previous reports [115, 116], the authors found that FABP1 is expressed in small intestine IECs but not in colon, and FABP1 mRNA and its protein (but not other members of the FABP family) were significantly reduced in DDX5-deficient small intestine IECs [69]. This indicates a high specificity in the DDX5-mediated regulation of FABP1. Their further studies indicated that DDX5 binds to and enhances FABP1 mRNA stability and ribosomal engagement in small intestine IECs (Fig. 7B) [69]. Given that DDX5^ΔIEC^ mice are resistant to small intestine tumorigenesis [69] and phenocopy the FABP1 KO mice reported previously [117], we can safely conclude that epithelial DDX5 promotes small intestine tumorigenesis at least partially due to DDX5-mediated post-transcriptionally enhancing FABP1 expression. Additionally, consistent with the fact that highly proliferative cells require large amounts of fatty acid building blocks from exogenous sources and/or de novo synthesis to sustain the building of cell membranes and organelles, regulation of FABP1 by DDX5 revealed a surprising role of DDX5 in intestinal lipid homeostasis (Fig. 7B) [69].

In summary, as demonstrated by Abbasi et al., DDX5-mediated tumorigenesis in colon is through controlling C3 expression, while DDX5-mediated tumorigenesis in small intestine is through controlling FABP1 expression [69]. Thus, while DDX5 has many downstream targets, DDX5 appears to have the ability to use different downstream targets in different tissue/cell types to accomplish its role in tumorigenesis. In other words, there are a lot of DDX5 upstream and downstream targets as previously reviewed [19]. However, which effector target(s) being used by DDX5 may be tissue/cell type-specific through DDX5 forming tissue and cell type-specific protein complexes with other partners as indicated by the authors [69]. This feature of DDX5 is intriguing. If more examples could be documented in the coming years, this would confirm a great advantage of the use of DDX5 as a target for cancer and/or other disease treatment, while avoiding potential toxicity to normal tissues during pharmaceutical inhibition or degradation of DDX5.

Excitingly, an important study from Le et al. revealed that (i) DDX5 is significantly enhanced in PCa tissues in comparison with benign prostatic hyperplasia [27]; (ii) high DDX5 is strongly associated with PCa progression and CRPC [27]; (iii) PCa patients with high DDX5 exhibited significantly shorter recurrence-free survival than patients with low DDX5 [27]; and (iv) use of antisense oligonucleotides (ASOs) inhibiting DDX5 significantly decreased PC-3 cell viability and delayed xenograft tumor growth [27]. Similarly, the study from Ling et al. found that (i) the small molecule FL118, acting as a molecular glue degrader, strongly binds to, dephosphorylates, and degrades DDX5 [46]; (ii) DDX5 acts as a master regulator to control the expression of multiple oncogenic proteins including survivin, Mcl-1, XIAP, cIAP2, c-Myc and mutant Kras (mKras) [46] (Fig. 1A); (iii) PDAC cells with DDX5 KO are resistant to FL118 treatment [46], and (iv) FL118 exhibits high efficacy to eliminate human colorectal and pancreatic cancer xenograft tumors that have high DDX5 expression, while FL118 exhibits less effectiveness for PDAC tumors with low DDX5 expression [46]. Additionally, by analysis of a large cohort of breast cancer (BC) tissues derived from 868 patients by using the immunohistochemistry (IHC) technology, the study from Li et al. revealed that (i) DDX5 is significantly overexpressed in BC tissues compared to adjacent normal tissues [118]; (ii) elevated DDX5 is associated with an aggressive phenotype in BC patients [118]; (iii) DDX5 is upregulated in recurrent patients compared with nonrecurrent patients, and DDX5 protein levels are positively associated with worse recurrence-free survival (RFS) and BC-specific survival (BCSS) in BC patients [118]; and (iv) high DDX5 expression in > 50-year old BC patients with advanced clinical stage or histological grade have a significantly increased risk of recurrence and shorter survival [118]. These authors concluded that their findings highlight the significance of DDX5 in the recurrence and clinical outcome of BC patients and DDX5 may be a potential predictive biomarker for patients with BC [118]. Together, these up-to-date studies have further strengthened the previous conclusion that DDX5 is a superior biomarker and target for cancer therapeutics [19].

**Fig. 8:**
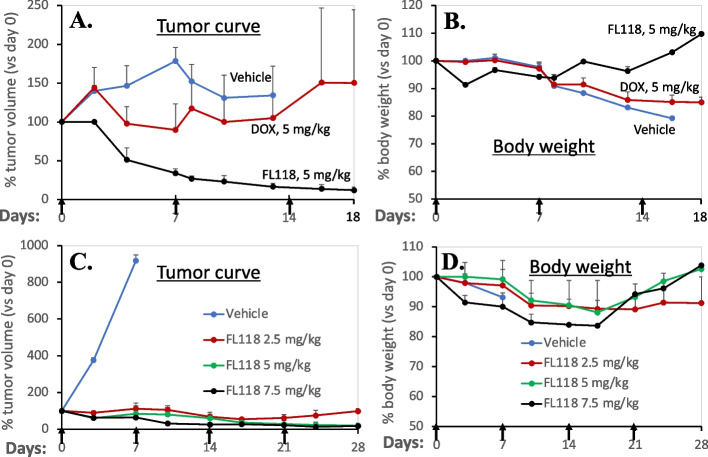
FL118 is potentially a superior anticancer drug against soft tissue sarcoma (STS): **A**, **B** FL118 (but not DOX) exhibited excellent efficacy against STS (**A**) with acceptable toxicity (**B**). The HT1080 STS cells (2 × 10^6^ per tumor site) mixed with 50% Matrigel were subcutaneously injected into 2–3 severe combined immunodeficiency (SCID) mice in the flank area to establish xenograft tumors. The established STS tumors were maintained on SCID mice. STS tumor SCID mice for the planned experimental studies were set up from the STS tumor‐maintained mice. Treatment with vehicle, DOX or FL118 at dose level of 5 mg/kg (DOX’s MTD – maximum tolerated dose) were started when tumors were grown into the size of 150—200 mm^3^. The schedule and route were weekly × 3 via intravenous (i.v.) administration (arrowed). **A** STS tumor change curves after vehicle, DOX and FL118 treatment. Each tumor curve is the mean tumor size + SD from 3 SCID mice. **B** Mouse body weight change curves after vehicle, DOX and FL118 treatment. Each body weight change curve is the mean body weight change + SD from 3 SCID mice. **C**, **D** FL118 exhibited high efficacy to regress STS tumors at FL118’s sub-MTD (**C**) with acceptable toxicity (**D**). HT1080 STS tumor establishment, experimental tumor mouse set up and treatment are the same as in A and B. The schedule and route were weekly × 4 via oral administration (arrowed). **C** STS tumor change curves after treatment with vehicle or FL118 at different dose levels as shown. Each tumor curve is the mean tumor size + SD from 3 SCID mice. **D** Mouse body weight change curves after treatment with vehicle or FL118 at different dose levels as shown. Each body weight change curve is the mean body weight change + SD from 3 SCID mice

Additionally, it is known that DDX5 is a multifunctional target and biomarker, and plays an important role in promoting cancer initiation, progression, metastasis and treatment resistance [19]. Studies indicate that this is not only for cancers like PDAC, CRC, PCa, etc. but also for several specialized rare cancer. Recent new publications further support the notion that DDX5 is a critical biomarker and target in specialized rare cancers. This includes (but may not be limited to) neuroblastoma [119], osteosarcoma [120, 121], rhabdomyosarcoma (RMS) [122, 123] and myeloproliferative neoplasms (MPN) [124]. These studies further demonstrate the advantage of using DDX5 as a target for anticancer drug development, since there are many special programs and mechanisms from the FDA and NIH to promote rare cancer drug development and commercialization. Consistent with these published studies, our studies indicated that the DDX5-targeting small molecule drug FL118 exhibits excellent efficacy against soft tissue sarcoma (STS), while the most used chemotherapy drug doxorubicin (DOX) for STS tumor treatment is not (Fig. 8).

### DDX5 as an emerging target in prostate cancer

Prostate cancer (PCa) is a heterogeneous malignancy that harbors diverse subpopulations of cancer cells with different phenotypes and biological functions [125]. PCa patients with low-grade tumors, i.e., those with combined Gleason Score (GS) of 6–7 are generally treated with radiation or radical prostatectomy with overall good prognosis. However, most patients diagnosed with high-grade PCa (GS 9–10) are frequently surgery-ineligible and treated with drugs that interfere with androgen receptor (AR) signaling. Thus, these drugs are called AR Signaling Inhibitors (ARSIs), which include androgen deprivation therapeutics (ADT, e.g., Lupron) and AR antagonists (e.g., enzalutamide). The majority of advanced PCa patients respond well to ARSIs at the beginning but most fail and become refractory to ARSI within ~ 2 years resulting in castration-resistant PCa (CRPC). Although many molecular mechanisms have been implicated in mediating and maintaining CRPC, one major cellular mechanism underlying CRPC development is the intrinsic cell heterogeneity and treatment-induced cellular plasticity [125]. For example, studies over the past several decades indicate that treatment-naïve PCa has both AR-expressing (AR^+^) and AR low-/non-expressing (AR^−/lo^) cancer cells as reviewed [126]. Recent work from the Tang lab [127] in human CRPC specimens and using both genetically engineered AR-null PCa cells and paired androgen-dependent (AD) and androgen-independent (AI) PCa xenograft models, demonstrates that while AR^+^ PCa cells, as expected, show exquisite sensitivity to enzalutamide, the AR^−/lo^ PCa cells are enzalutamide-resistant de novo. Thus, AR^−/lo^ PCa cells are inherently resistant to ARSIs and AR^−/lo^ metastatic CRPC (mCRPC) represents a more aggressive and lethal subtype of PCa.

**Fig. 9:**
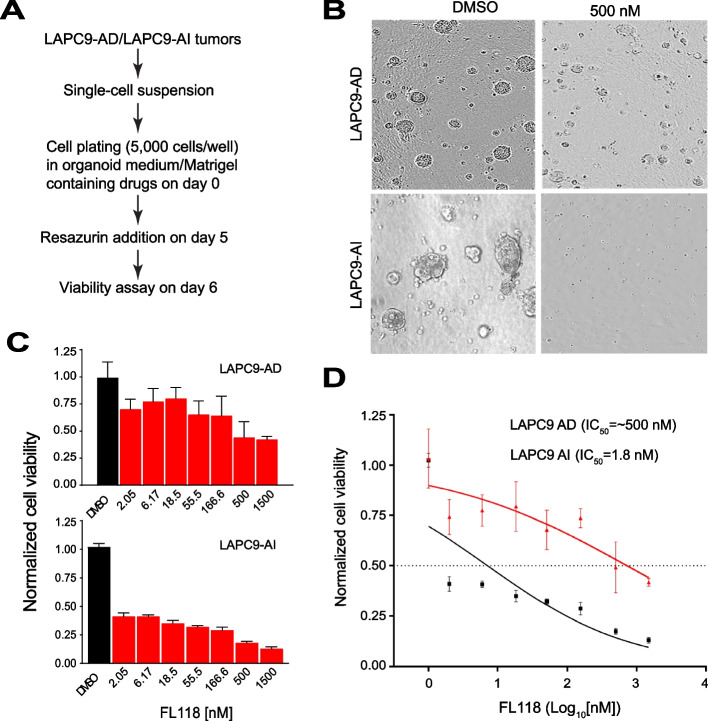
FL118 preferentially inhibits AR^−/lo^ LAPC9-AI cells in organoid screening assays:** A** Experimental schema. LAPC9-AD/AI cells were purified out from the maintenance tumors. Cytotoxic/cytostatic effects of drug (FL118) treatment were measured using resazurin assays in triplicate culture (5,000 cells/well in 96-well plate).** B** Representative LAPC9-AD and LAPC9-AI organoid images 6 days after FL118 treatment.** C**,** D** FL118 exhibited a more prominent inhibitory effect on AR^−/lo^ LAPC9 than AR.^+^ LAPC9-AD organoids. Shown in C are bar graphs of LAPC9-AD (top) and LAPC9-AI (bottom) organoids in the presence of vehicle control (DMSO) or increasing concentrations of FL118. Data are normalized to DMSO-treated control samples and presented as mean ± SD. Shown in D are the dose–response curves generated using relative viable cell numbers after treatment with increasing concentrations of FL118 for 4 days. The inhibitory effects (IC_50_) of FL118 on LAPC9-AI compared to LAPC9-AD are statistically highly significant (*P* < 0.0001; two-way ANOVA)

**Fig. 10:**
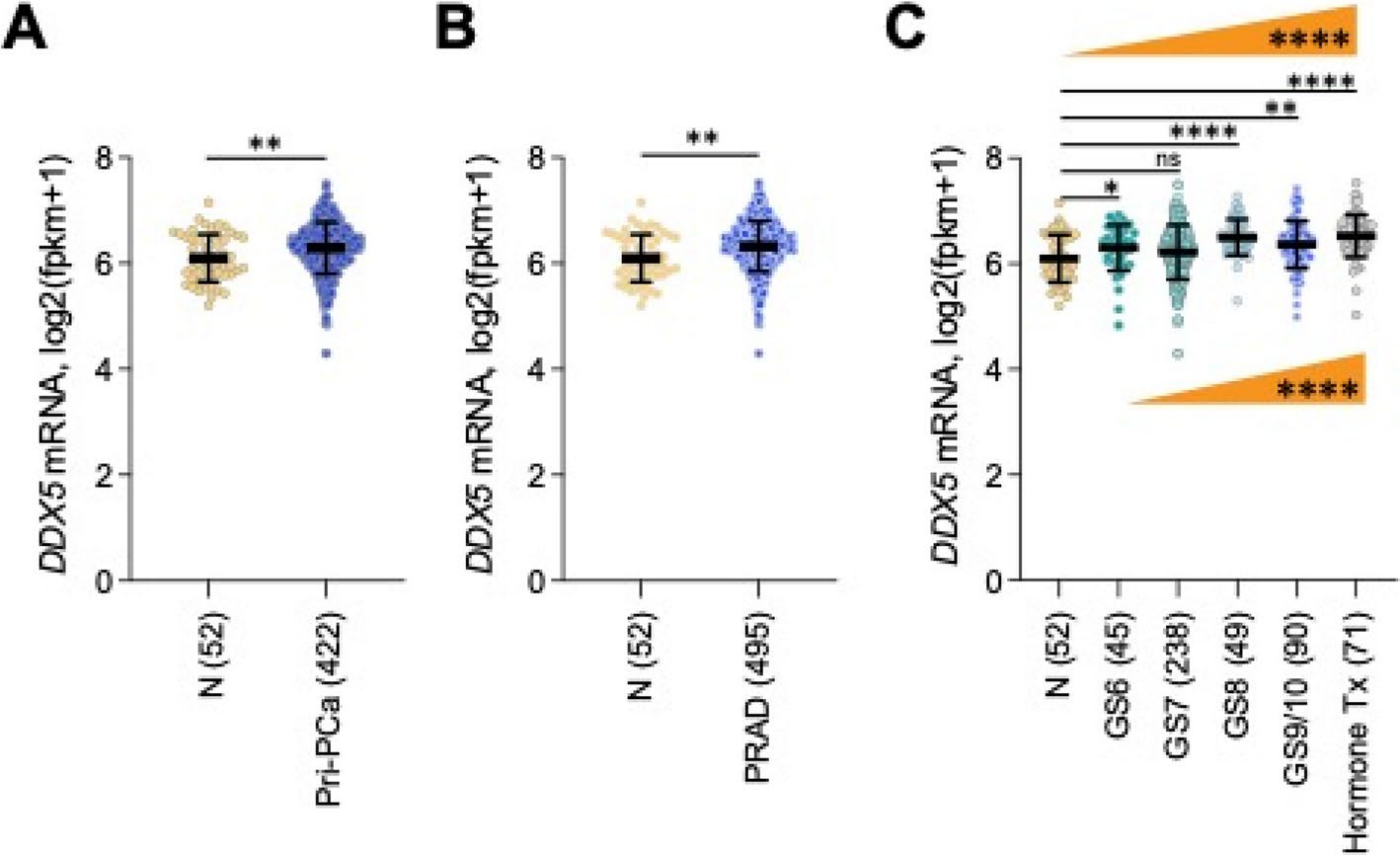
*DDX5* mRNA levels are upregulated in PCa and correlate with tumor grade:** A**, **B** Increased *DDX5* mRNA levels in human PCa. Shown are *DDX5* mRNA levels in two different tumor-normal (N) comparisons from the TCGA-PRAD dataset, i.e., 422 treatment-naive PCa (Pri-PCa; A) and 495 total PCa patients in PRAD which includes the 422 Pri-PCa as well as PCa treated with hormone therapy [71] and chemotherapy [2]. **P < 0.01 (Student’s* t*-test).** C** Increasing *DDX5* mRNA levels in human PCa correlate with tumor grade as indicated by increasing Gleason Scores (GS). *DDX5* mRNA levels and corresponding PCa patients’ tumor grade were extracted from UCSC Xena database (http://xena.ucsc.edu/). Data is presented as mean ± SD. Statistical analyses were performed using GraphPad Prism software or using R. Student’s *t*-test was used to compare PCa of various grades to N while Jonckheere-Terpstra’s trend (J-T) test was used to calculate the statistical significance of the trend across all different groups. *P < 0.05; **P < 0.01; **** *P* < 0.0001

A top priority is to identify and develop novel therapeutic agents or combinations to target AR^−/lo^ PCa cells and CRPC. In this regard, it is of great interest that FL118, a potent small-molecule anticancer drug developed in the Li lab, exhibited selective toxicity against the AR^−/lo^ LAPC9-AI vs. AR^+^ LAPC9-AD cells in organoid assays (Fig. 9). Briefly, we purified LAPC9-AD and LAPC9-AI cells from the respective maintenance tumors and performed quantitative organoid assays (Fig. 9A). FL118, in a dose-dependent manner, inhibited LAPC9-AI organoids more prominently than the LAPC9-AD organoids (Fig. 9BCD). It is as of yet unclear whether FL118’s selective toxicity to AR^−/lo^ LAPC9-AI cells depends on DDX5, which has been shown to be bound and degraded by FL118 [46]. Excitingly, we found that DDX5 is significantly upregulated in human PCa compared to the adjacent benign/normal prostatic tissue (Fig. 10AB). Notably, the upregulated *DDX5* mRNA levels in PCa correlate with increasing tumor grade, both when compared with the normal tissue and when compared among the tumors of varying grade (Fig. 10C). These results support an oncogenic role of DDX5 in PCa. Our observations also suggest that the preferential inhibitory effects of FL118 on AR^−/lo^ LAPC9-AI cells may be mediated, at least in part, via DDX5, which is expressed at higher levels in high-grade advanced PCa, which are enriched in AR^−/lo^ PCa cells [125–127].

There have been a few reports on DDX5 expression and functions in PCa. DDX5 was first reported to be fused in frame with ETV4 leading to the expression of DDX5-ETV4 fusion protein [128]. DDX5 is not an androgen-regulated or androgen-induced gene; instead, DDX5 may function as an AR transcriptional co-activator thus promoting expression of AR target genes [129]. Interestingly, both DDX5 and β-catenin are overexpressed in advanced high-grade prostate tumors and the two physically interact in both androgen-dependent and -independent manners [8]. DDX5 was reported to be a direct protein binder of resveratrol, the claimed beneficial chemopreventive molecule enriched in red wine, and resveratrol binding to and inducing degradation of DDX5 [130]. DDX5 also functions as a coactivator of Wnt activator FOXB2 driving the development of advanced and neuroendocrine-like PCa [131]. DDX5 may also modulate AR-regulated mRNA alternative splicing [132]. Notably, a recent study has reported DDX5 interactions with the Ku70/Ku8 heterodimers and implicated DDX5 in DNA damage repair in PCa [27]. Overall, these studies have linked DDX5 to promoting PCa development and progression. Indeed, downregulation of DDX5 by ASO inhibited PCa cell proliferation and overcame CRPC resistance [27], supporting that DDX5 may represent a good therapeutic target for the treatment of aggressive PCa.

### Reciprocal function of DDX5 in normal tissues results in favorable toxicology profiles

DDX5 was shown to promote the progression of acute myeloid leukemia (AML), and DDX5 inhibition can induce apoptosis of AML cells without any toxicity to bone marrow cells [78]. Even 90% of DDX5 KD in the bone marrow did not induce detectable apoptosis or impair bone marrow function [78], leading to the conclusion that DDX5 is dispensable for or not involved in normal hematopoiesis and tissue homeostasis [78]. Several other studies support this conclusion: ASO-mediated KD of DDX5 in mouse tissues showed no toxicity [27] and FL118 is very well tolerated in mice and dogs [133]. Such low toxicity of DDX5 inhibition in normal tissues can be explained by the observation that DDX5 has opposite function in normal cells/tissues versus in cancer cells/tissues: In contrast to the involvement of DDX5 in tumorigenesis, cancer cell proliferation, migration and metastasis [18], DDX5 was recently shown to inhibit smooth muscle cell (SMC) proliferation [134]. DDX5 KD or KO increased SMC proliferation and migration, whereas overexpression of DDX5 prevented proliferation and migration of SMCs [134]. SMC DDX5-deficient mice show exacerbated neointima formation after femoral artery injury, while DDX5 overexpression potently inhibited vascular remodeling in balloon-injured rat carotid arteries [134]. Furthermore, DDX5 suppresses IIR activation by (i) promoting IL-1β production and recruiting neutrophils into TME [64], (ii) inhibiting the production of IFN-β [65] and (iii) augmenting the expression of immunosuppressive factor CD24 [66], while DDX5 restricts RORγt + T_regs_ suppressor function to promote intestine inflammation [68]. All of these observations suggest that pharmaceutical inhibition or degradation of DDX5 in normal tissues/cells or immune cells may not only show little toxicity but may even benefit healthy tissues while being selectively active against tumorigenesis.

However, such a favorable role of DDX5 may not apply to all normal tissues, since DDX5 is also involved in spermatogenesis through regulating gene expression programs and activity of undifferentiated spermatogonia in mice [135]. Consistently, another study found a similar role of DDX5 in neonatal mouse gonocyte survival [136]. The authors demonstrated that (i) germ cell-specific DDX5 KO (DDX5-/-) leads to infertility in adult male mice due to the complete elimination of germ cells [136]; (ii) male germ cells gradually disappeared in DDX5(-/-) mice from E18.5 to P6 [136]; and (iii) DDX5 ablation impeded the proliferation of gonocytes [136]. However, in zebrafish, the majority of DDX5-deficient zebrafish developed as fertile males with normal testes and a small number of DDX5-deficient zebrafish developed as infertile females with small ovaries [137]. This supports the notion that DDX5 is dispensable for testis development, but it is essential for female sex differentiation and oocyte maturation in zebrafish [137]. These differences found in the studies [136, 137] may reflect another example that DDX5 has the ability to select different partners to differentiate in its regulation features.

In summary, DDX5 emerges as an ideal target for the treatment of cancer. The side effect of inhibition of either sperm or oocyte formation during the DDX5-targeting cancer treatment is not a major concern as the majority of effected cancer patients are over 50s years of age. Since the inhibition of DDX5 does not induce smooth muscle cell loss; instead, increasing their proliferation and survival [134], this effect can additionally benefit cancer patients.

### Concluding remarks

DDX5 is emerging as an attractive target for cancer therapeutic development. DDX5 involves diverse oncogenic signaling pathways including cancer-controlled gene expression, various RNA metabolism, DNA repair and R-loop resolution during DNA replication and transcription (Figs. 1 and 2), immune suppression (Fig. 3), cancer metabolic control (Fig. 4), virus infection promotion (Fig. 5), and microbiota negative influence (Fig. 6). Virus infection and bad microbiota in gut are known to be involved in helping cancer initiation and progression [87, 88, 97]. Importantly, DDX5 uses distinct signaling cascades by interacting with different proteins in different tissues/cells (Fig. 7) [69] as well as in normal tissues/cells versus in cancer tissues/cells to elicit distinct roles in healthy [134] versus in cancer cells [18]. Consistently, our studies indicated that while FL118 shows excellent anti-sarcoma tumor activity (Fig. 8), FL118 exhibits selective toxicity against the AR^−/lo^ LAPC9-AI vs. AR^+^ LAPC9-AD PCa cells in PDAC-derived organoids (Fig. 9). These unique characteristics of DDX5 make DDX5 a particularly interesting target for the treatment of cancer and potentially, for other diseases as well.

**Fig. 11:**
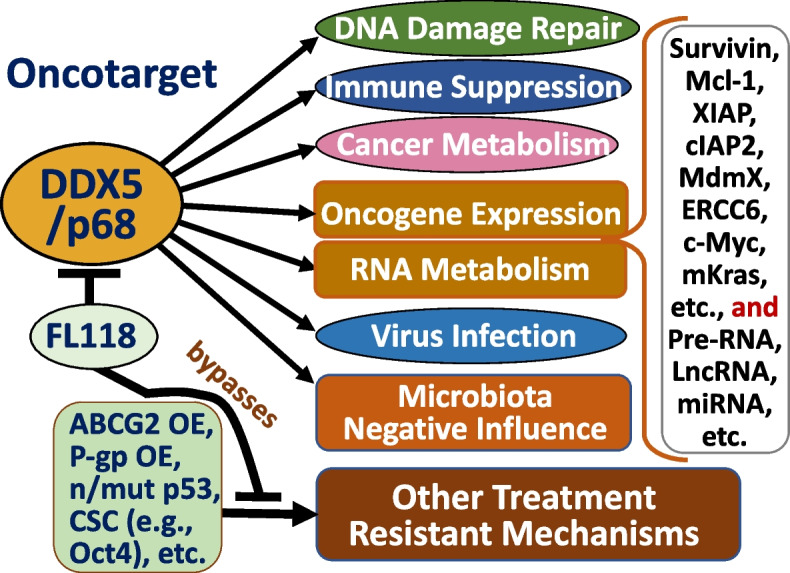
DDX5 (also called p68 in early studies) plays important roles in multiple treatment resistant mechanisms: This includes, but may not be limited to, promoting DNA repair and R-loop resolution during DNA replication and gene transcription (this review); promoting immune suppression (this review); controlling cancer metabolism (this review); promoting oncogene expression (e.g., survivin, Mcl1, XIAP, cIAP2, MdmX, ERCC6, c-Myc, mutant Kras (mKras), etc., which can be indirectly inhibited by FL118 [46, 133, 139, 140]); controlling various types of RNA metabolism (e.g., pre-RNA, long and short/small non-coding RNA) and ribosome biogenesis [141]; promoting virus infection and replication (this review); and negatively influences microbiota (this review). On the other hand, while FL118 targets DDX5, FL118 could also bypass many other treatment resistant mechanisms (e.g., overexpression (OE) of ABCG2/BCRP [142, 143] and/or OE of P-gp/MDR-1 [142]; null/mutated p53 (n/mut p53) [140]; cancer stem cell (CSC)-induced treatment resistance [144], etc.)

As reviewed in the section of “Reciprocal Function of DDX5 in Normal Tissues Results in Favorable Toxicology Profiles” as well as in other sections, targeting DDX5 produced favorable toxicology profile and could shift cancer cells from either the proliferative or growth-arrested state into the apoptotic and cancer cell killing states. Thus, a small molecule-based “molecular glue degrader” [138], such as FL118 [46], may represent a particularly valuable and convenient approach to treat DDX5-dependent malignancies (which are linked to multiple treatment resistant mechanisms), especially those that are implicated with other treatment resistant mechanisms as shown in Fig. 11.

## Data Availability

Refer to the information provided in the Review article.

## Abbreviations

AR: Androgen receptor
AREG: Amphiregulin
CRC: Colorectal cancer
CRPC: Castration-resistant prostate cancer
DSBs: Double-strand breaks
EMT: Epithelial mesenchymal transition
G4: G-quadruplexes
HBV: Hepatitis B virus
HCC: Hepatocellular carcinoma
HIF1α: Hypoxia-induced factor 1α
HR: Homologous recombination
HXTs: Hexose transporters
IAV: Influenza A virus
IFN: Interferon
IIRs: Innate immune responses
iPSCs: Induced pluripotent stem cells
ISCs: Intestinal stem cells
ISGs: IFN-stimulated genes
KD: Knockdown
KO: Knockout
MDV: Marek's disease virus
mKras/mKRAS: Mutant Kras/KRAS
ncRNA: Non-coding RNA
PCa: Prostate cancer
PDAC: Pancreatic ductal adenocarcinoma
RH: RNA helicase
RPA: Replication protein A
SMC: Smooth muscle cell
STING: Stimulator of Interferon Genes
TANs: Tumor-associated neutrophils
TF: Transcription factor
TME: Tumor microenvironment
WT: Wild type
